# Transcriptomic changes during caste development through social interactions in the termite *Zootermopsis nevadensis*

**DOI:** 10.1002/ece3.4976

**Published:** 2019-02-23

**Authors:** Hajime Yaguchi, Ryutaro Suzuki, Masatoshi Matsunami, Shuji Shigenobu, Kiyoto Maekawa

**Affiliations:** ^1^ Graduate School of Science and Engineering University of Toyama Toyama Japan; ^2^ Tropical Biosphere Research Center University of the Ryukyus Nishihara Japan; ^3^ Graduate School of Medicine University of the Ryukyus Nishihara Japan; ^4^ Functional Genomics Facility National Institute for Basic Biology Okazaki Japan

**Keywords:** caste differentiation, Gene Ontology, Kyoto Encyclopedia of Genes and Genomes, RNA‐seq, social interaction

## Abstract

One of the most striking examples of phenotypic plasticity is the different phenotypes (i.e., castes) within a same nest of social insects. Castes usually derive from a single genotype initially by receiving social cues among individuals during development. Specific gene expression changes may be involved in caste differentiation, and thus, the regulatory mechanism of these changes should be clarified in order to understand social maintenance and evolution. The damp‐wood termite *Zootermopsis nevadensis* is one of the most important model termite species, due to not only the availability of genomic, transcriptomic, and epigenomic information but also evidence that soldier‐ and worker‐destined individuals can be identified in natural conditions. Given that the nutritional intakes via social interactions are crucial for caste differentiation in this species, there is a possibility that transcriptomic changes are influenced by the nutritional difference among these individuals. Here, whole body RNA‐seq analysis of 3rd‐instar larvae with biological replications and Gene Ontology and Kyoto Encyclopedia of Genes and Genomes enrichment analyses were conducted. We found the drastic expression differences during caste developments between soldier‐ and worker‐destined individuals. The results indicated that there are several key signaling pathways responsible for caste formations, which are involved in developments and social interactions. Particularly, the nutritional sensitive signaling was upregulated in soldier‐destined individuals, while some metabolic pathways were identified in worker‐destined individuals. These bioinformatic data obtained should be utilized to examine the molecular mechanisms of caste determination in social insects.

## 1 INTRODUCTION

One of the major goals in ecological developmental biology is to reveal the molecular mechanisms underlying phenotypic plasticity, which allows organisms to produce different phenotypes from the same genomic background depending on environmental cues (Gilbert & Epel, 2009). Phenotypic plasticity is particularly widespread in insects (Simpson, Sword, & Lo, 2011), and for social insects (e.g., bees, wasps, ants, and termites), it is a crucial for their social organization and evolutionary success (Wilson, 1971). In a colony of social insects, there are multiple phenotypes (i.e., castes) sharing the same genomic background, and they have specialized morphologies and behaviors to achieve their respective roles. Such species are an excellent resource to study the molecular mechanisms of phenotypic plasticity, because the complex social signals as well as environmental cues usually impact on caste differentiation during postembryonic development (Linksvayer, Fewell, Gadau, & Laubichler, 2012).

Termite is one of the most successful social insects, and their social organization was acquired independently from hymenopteran insects. Termite castes are normally composed of reproductives, workers, and soldiers, and unlike the social Hymenoptera, the soldier caste was first acquired as a permanently sterile caste during social evolution (Nalepa, 2011; Tian & Zhou, 2014). Soldiers are differentiated from workers via an intermediate presoldier stage. The new production of soldiers is promoted by the presence of reproductives (Bordereau & Han, 1986; Maekawa, Nakamura, & Watanabe, 2012) and suppressed by existing soldiers (Mitaka, Mori, & Matsuura, 2017; Watanabe, Gotoh, Miura, & Maekawa, 2011). Caste differentiation is generally determined in an environmentally sensitive period during postembryonic development, probably by pheromonal substrates transmitted via inter‐individual interactions (Noirot, 1991; Watanabe, Gotoh, Miura, & Maekawa, 2014). Consequently, changes in gene expression caused by the social interactions should be clarified to understand the molecular mechanisms underlying caste differentiation.

The damp‐wood termite *Zootermopsis nevadensis* is useful taxon for sociogenomic studies, because genomic, transcriptomic, and epigenomic information is available (Glastad, Gokhale, Liebig, & Goodisman, 2016; Terrapon et al., 2014). Importantly, in this species soldier‐destined individuals can be identified in natural conditions (Maekawa et al., 2012; Masuoka, Yaguchi, Suzuki, & Maekawa, 2015; Yaguchi, Inoue, Sasaki, & Maekawa, 2016). In an incipient colony, the first soldier was differentiated from the first‐molted 3rd‐instar larva (hereafter No. 1 larva). On the other hand, workers were molted from the 2nd (No. 2) or subsequent molted 3rd‐instar larva. The No. 1 larva always differentiated into a presoldier after 7–8 days, whereas the No. 2 larva normally molted into a 4th instar after approximately 20 days and functioned as a worker. The No. 2 larvae could differentiate into a soldier, when the No. 1 larva was artificially removed from the incipient colony, suggesting that soldier differentiation was determined by environmental conditions (Maekawa et al., 2012). Importantly, proctodeal trophallaxis (i.e., anal feeding) from the reproductives to the No. 1 larvae was more frequently observed than to the No. 2 larvae, but allogrooming of the No. 2 larvae was more frequently observed than that of the No. 1 larvae. These behavioral differences initially occurred at Day 1 or 2 after their appearance (Maekawa et al., 2012). These observations suggested that the nutritional conditions were apparently different between these larvae. Developments of the No. 1 larvae typically depended on the unique food intakes from reproductives, but those of the No. 2 larvae were required mainly for self‐feeding of woods. Therefore, there is a possibility that the caste formations are influenced by the intrinsic factors in response to the nutritional differences between these larvae. There are, however, no molecular evidences to support these developmental changes.

Generally, soldier differentiation in termites requires an increase in juvenile hormone (JH) titer in workers (Miura & Scharf, 2011). In *Z. nevadensis*, JH biosynthetic genes in the No. 1 larvae were highly expressed from the period beyond 3 days after their appearance (Yaguchi, Masuoka, Inoue, & Maekawa, 2015). Moreover, a specific expressed lipocalin gene (*Neural Lazarillo*; *NLaz*) was identified in the No. 1 larva at day 3 after the appearance (Yaguchi et al., 2018). A lipocalin gene *ZnNLaz1 *was a crucial regulator for solder differentiation through the regulation of trophallactic interactions with a queen. These results suggested that there was a cross talk between lipocalin and the intrinsic factors to integrate the social signals including unique nutrient intakes from a queen. Thus, soldier‐ and worker‐destined larvae, that is, the No. 1 and No. 2 larvae, especially within 3 days after their appearance, are important materials to examine the gene expression patterns for the behavioral and physiological changes involved in caste differentiation.

Here, we revealed the transcriptomic changes in response to the nutritional differences underlying caste differentiation in *Z. nevadensis*. By performing RNA‐seq analysis, large numbers of differentially expressed genes (DEGs) were identified in both the No. 1 and No. 2 larvae. Functional significance of DEGs was inferred using Gene Ontology (GO) database, and Kyoto Encyclopedia of Genes and Genomes (KEGG) database. Based on the results obtained using these bioinformatic analyses, we discuss the molecular basis underlying caste differentiation in termites.

## 2 MATERIALS AND METHODS

### 2.1 Incipient colony foundation of *Zootermopsis nevadensis*

Mature colonies (total 11) of the damp‐wood termite *Z. nevadensis* were collected from Kawanishi‐shi, Hyogo Prefecture, Japan, in April and June 2015. The collected colonies were placed in plastic containers and maintained in consistent darkness at room temperature in the laboratory until the emergence of alates (winged adults). Alates were collected from these colonies, and the sexes of individuals were confirmed using the morphology of abdominal sternites (Weesner, 1969). The incipient colonies were established with unrelated alates from 11 different mature colonies in accordance with previous reports (Maekawa et al., 2012; Yaguchi et al., 2016, 2015). The constructed 48 incipient colonies (Supporting Information Table S1) were kept in consistent darkness at 25°C in an incubator. After the appearance of the 2nd‐larval instar, the colonies were checked carefully every 24 hr to confirm whether a 3rd‐instar larva had appeared. The oldest 3rd‐instar larva (No. 1 larva = soldier‐destined larva) and the second 3rd‐instar larva (No. 2 larva = worker‐destined larva) at Day 0 after their appearance were marked with different colored ink spots to discriminate each larva. The two respective larvae were collected at Day 1 (*n* = 6), 2 (*n* = 6), and 3 (*n* = 12), all of which were individually sampled from the different colonies (Supporting Information Table S1). The No. 2 larvae were collected at the periods when the presoldier exists in the colony, and thus, they were definitely worker‐destined larvae. The collected larvae were immersed immediately in liquid nitrogen and stored in −80°C for the following experiments.

### 2.2 Total RNA extraction, RNA‐seq library preparation, and sequencing by HiSeq1500

Total RNA was extracted from whole body (4 individuals were used for each library) of the No. 1 and No. 2 larvae using a SV Total RNA extraction kit (Promega, Madison, WI, USA). Extracted total RNA from larvae at Day 1–2 was mixed equally (2 individuals from each day; Supporting Information Table S1). The amounts of RNA and DNA in each sample were quantified using a Qubit fluorometer (Life Technology, Eugene, OR, USA), and the qualities of those were confirmed using an Agilent 2100 bioanalyzer (Agilent Technologies, Palo Alto, CA, USA). Total RNA (500 ng) was used for cDNA synthesis and purification based on a low‐throughput protocol with a TruSeq Stranded RNA LT kit (Illumina, San Diego, CA, USA). A half‐scale reaction of the standard protocol was applied for library preparation. The quality and quantity of cDNA were validated using an Agilent 2100 bioanalyzer and a KAPA qPCR SYBR green PCR kit (GeneWorks, Thebarton, Australia). RNA‐seq analysis was performed by single‐end sequencing (66 bp) using Hiseq1500 (Illumina, San Diego, CA, USA). Three replicates (i.e., biological triplicates) were prepared for four different developmental stages (No. 1 larvae at Day 1–2, No. 2 larvae at Day 1–2, No. 1 larvae at Day 3, and No. 2 larvae at Day 3), and a total of 12 libraries were sequenced (Supporting Information Table S1). All reads have been deposited in the DDBJ Sequence Read Archive (DRA) database under accession numbers DRA007363.

### 2.3 Identification of differentially expressed genes between soldier‐ and worker‐destined larvae

**Figure 1:**
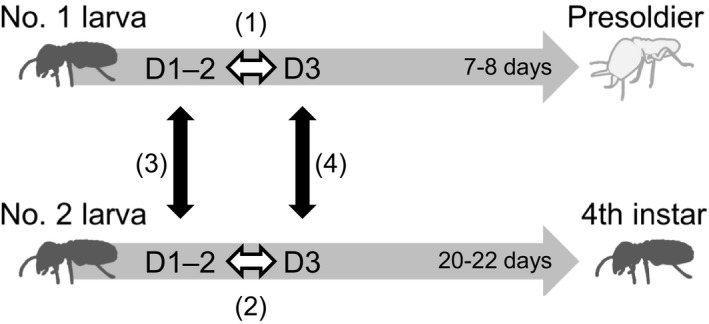
Experimental scheme of RNA‐seq analysis between soldier‐ and worker‐destined larvae collected from incipient colonies of *Zootermopsis nevadensis*. The first‐molted 3rd‐instar larva (No. 1 larva) always differentiates into a presoldier, and the second‐molted 3rd‐instar larva (No. 2 larva) molts into a 4th instar and functions as workers in the colony. The No. 1 and No. 2 larvae were collected at Day 1–2 (D1–2) and Day 3 (D3).

Prior to mapping of the transcriptomic data, all libraries were processed in order to perform expression analyses as follows. Firstly, the read quality of the obtained sequence reads was checked using FastQC (Andrews, 2010), and the adaptor sequences were removed from all libraries using cutadapt 1.4.2 (Martin, 2011) with default parameters. The trimming of low‐quality reads was performed using SolexaQA v2.5 (Cox, Peterson, & Biggs, 2010) with a Phred score cutoff of 28 (‐h 28) in DynamicTrim.pl and a minimum trimmed read length of 23 (‐l 23) in LengthSort.pl. These reads were mapped to a *Z. nevadensis* reference genome (gene model OGSv2.2; Terrapon et al., 2014) using TopHat v2.0.9 (Kim et al., 2013) with default parameters. Counting of reads was performed using featureCounts v1.5.2 (Liao, Smyth, & Shi, 2014). Differential gene expression levels were compared using a generalized linear model (GLM) approach implemented using the edgeR 3.18.1 Bioconductor package (Robinson, McCarthy, & Smyth, 2010). Normalization factors for each library were calculated using the trimmed mean of *M*‐values (TMM) method (Robinson & Oshlack, 2010). A multidimensional scaling (MDS) plot was described using the normalized count data of all libraries in R v.3.4.0 (R Core Team, 2017). Four pairwise comparisons were performed: (a) Day 1–2 × Day 3 in the No. 1 larva, (b) Day 1–2 × Day 3 in the No. 2 larva, (c) the No. 1 larva × No. 2 larva at Day 1–2, and (d) the No. 1 larva × No. 2 larva at Day 3 (Figure 1). MA plots were used to represent the difference in counts in each developmental stage by edgeR. A false discovery rate (FDR) <0.05 was used to the cutoff of differentially expression. Furthermore, ANOVA‐like comparisons were performed to know the caste‐biased genes with age effects using edgeR with GLM analysis (McCarthy, Chen, & Smyth, 2012).

### 2.4 Gene Ontology and Kyoto Encyclopedia of Genes and Genomes enrichment analysis

To understand the different functional profiles between the No. 1 and No. 2 larvae, GO and KEGG enrichment analyses were performed. For those analyses, we assigned an ortholog of *Drosophila melanogaster* for each *Z. nevadensis* gene, based on reciprocal best hits between *Z. nevadensis* genes (gene model OGSv2.2; Terrapon et al., 2014) and *D. melanogaster *genes (available from FlyBase, version: FB2018 r6.23). BlastP searches were carried out for 15,876 *Z. nevadensis* genes and 30,506 *D. melanogaster *protein sequences (version: FB2018 r6.23) with default parameters. The reciprocal best hit genes showing less than *e*‐value 1.0*e*−50 were assigned as the orthologs. Of the assigned 5,424 genes, DEGs with more than twofold changes (total 800 genes) were used for GO and KEGG enrichment analyses performed using an R package clusterProfiler (Yu, Wang, Han, & He, 2012) with fly annotations (Carlson, Pagès, Arora, Obenchain, & Morgan, 2016). The GO enrichment analysis was performed focused on biological process. *p*‐Values were adjusted using the multiple test correction method of Benjamini and Hochberg (Benjamini & Hochberg, 1995).

## 3 RESULTS

### 3.1 Overall transcriptomic profiles

**Figure 2:**
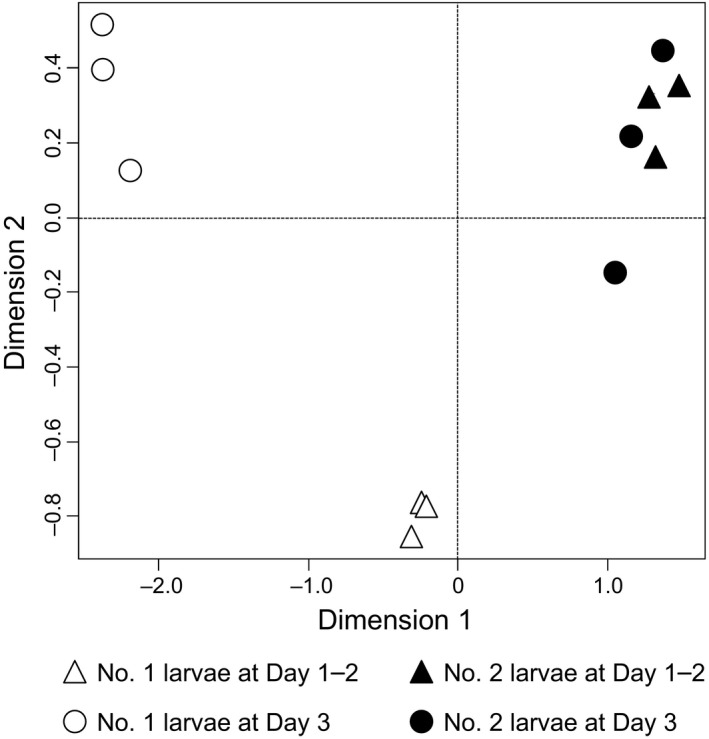
Multidimensional scaling (MDS) plots of all genes detected in the RNA‐seq data. White and black marks indicate soldier‐destined (No. 1) and worker‐destined (No. 2) larvae, respectively. RNA‐seq libraries obtained from larvae at Day 1–2 and Day 3 are represented as triangles and circles, respectively

We investigated overall gene expression profiles from whole bodies of soldier‐destined (No. 1) and worker‐destined (No. 2) larvae at Day 1–2 and Day 3. Sequencing using Illumina HiSeq1500 platform yielded 138.9 million 66‐bp single‐end sequence reads (Supporting Information Table S2). To evaluate the transcriptomic differences among the No. 1 and No. 2 larvae at both time points after their appearance, the MDS plot was described using normalized count data calculated for each library. Each replication was clustered and similar to each other (Figure 2). The MDS plot showed that the overall transcriptome was differentially plotted between Day 1–2 and Day 3 in the No. 1 larvae. However, no obvious differences were observed between Day 1–2 and Day 3 in the No. 2 larvae relative to those of the No. 1 larvae.

### 3.2 Differentially expressed genes between each developmental stage

**Figure 3:**
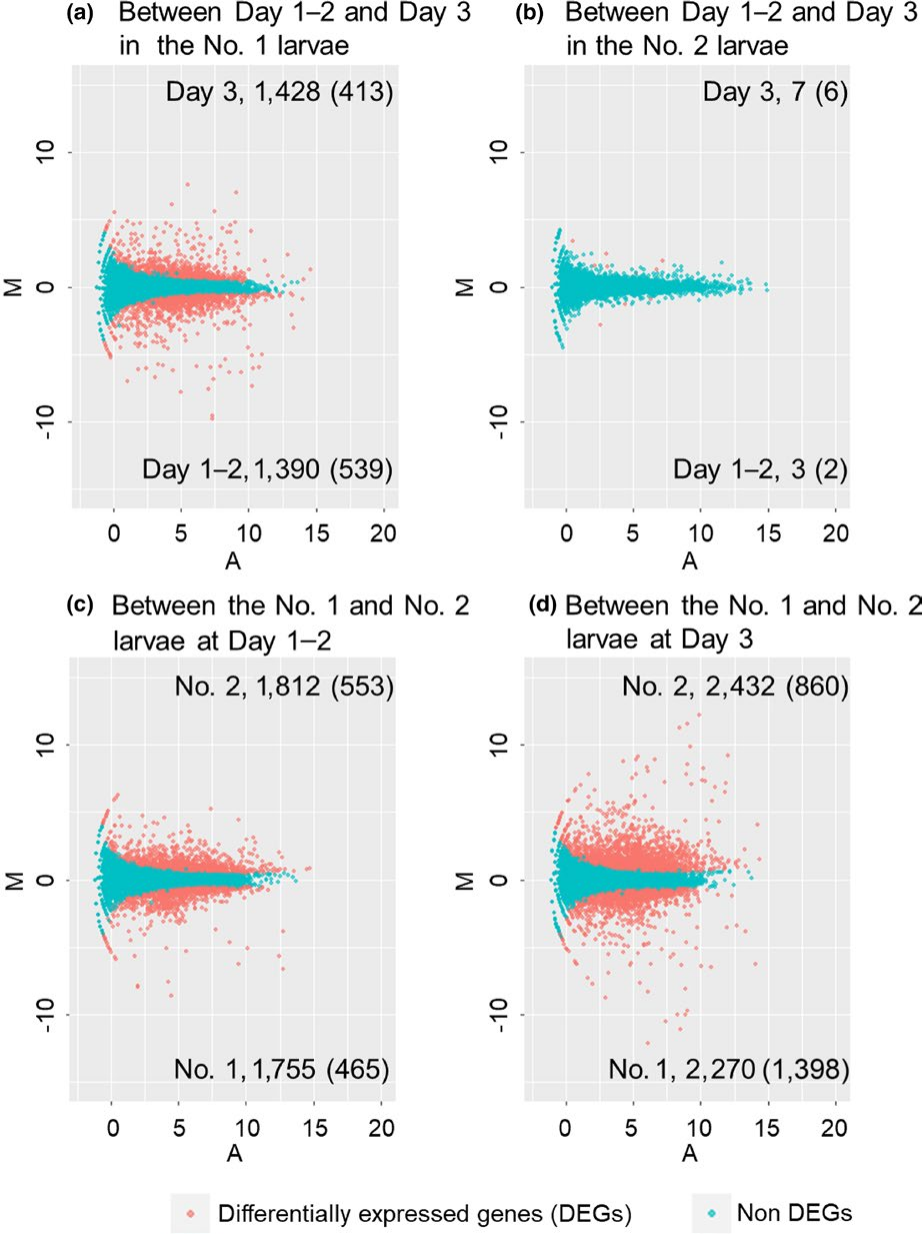
MA plots of RNA‐seq data. The *x*‐axis (A) indicates a log mean value of counts per millions (CPM). The *y*‐axis (M) indicates a log fold value. The color plots show significantly (red) and nonsignificantly (blue) differentially expressed genes (FDR < 0.05). Numbers in a graph show those of upregulated genes in each pairwise comparison. Numbers in parenthesis show the number of genes with fold changes above twofold in their expression levels. The pairwise comparison between Day 1–2 and Day 3 in the No. 1 larvae (a), between Day 1–2 and Day 3 in the No. 2 larvae (b), between the No. 1 and No. 2 larvae at Day 1–2 (c), and between the No. 1 and No. 2 larvae at Day 3 (d).

We obtained different numbers of DEGs for the four pairwise comparisons (Figure 3). The number of DEGs between Day 1–2 and Day 3 in soldier‐destined (No. 1) larvae was 2,818. Of these genes, 1,390 and 1,428 genes were upregulated in Day 1–2 and Day 3, respectively (Figure 3a and Supporting Information Tables S3 and S4). By contrast, there were only 10 DEGs between Day 1–2 (three genes were upregulated) and Day 3 (seven genes were upregulated) in worker‐destined (No. 2) larvae (Figure 3b and Supporting Information Tables S5 and S6). The expression levels of 3,567 genes were significantly different between the No. 1 and No. 2 larvae at Day 1–2. Of these 3,567 DEGs, 1,755 and 1,812 genes were upregulated in the No. 1 and No. 2 larvae, respectively (Figure 3c and Supporting Information Tables S7 and S8). The expression levels of 4,702 genes were significantly different between the No. 1 and No. 2 larvae at Day 3. Of these 4,702 DEGs, 2,270 and 2,432 genes were upregulated in the No. 1 and No. 2 larvae, respectively (Figure 3d and Supporting Information Tables S9 and S10). The *ZnNLaz1* (gene ID: Znev_05665) was highly expressed in the No. 1 larvae at Day 3 relative to the No. 2 larvae at Day 3 (Supporting Information Table S9). In the ANOVA‐like comparisons, caste‐biased genes with age effects were detected between the No. 1 and No. 2 larvae (Supporting Information Figure S1). In the No. 1 larvae, 932 and 1,723 genes were highly expressed at Day 1–2 and Day 3, respectively (Supporting Information Figure S1 and Tables S11 and S12). On the other hands, 1,859 and 905 genes were highly expressed at Day 1–2 and Day 3 in the No. 2 larvae, respectively (Supporting Information Figure S1 and Tables S13 and S14). Again, the *ZnNLaz1* (Znev_05665) was highly expressed in the No. 1 larvae at Day 3 (Supporting Information Table S12).

### 3.3 Gene Ontology enrichment analysis in DEGs

GO enrichment analyses (Yu et al., 2012) were performed using the identified DEGs with more than twofold changes for each pairwise comparison (Figure 3) in order to reveal the molecular signatures for caste differentiation. The enriched GO terms (biological process) were identified among the DEGs between each developmental stage (Supporting Information Figure S2, Tables S15–S20).

Using 539 DEGs upregulated in soldier‐destined (No. 1) larvae at Day 1–2 relative to Day 3 (Figure 3a), there were 28 enriched GO terms, which were related to metabolic process as well as cuticle development (Supporting Information Figure S2, Table S15). In particular, GO terms related to nutritional substrates were identified, such as lipid (GO:0006629, 0008610, and 0055088) and fatty acids (GO:0006631, 0006633, 0030497, and 0000038). On the other hand, using 413 DEGs upregulated in soldier‐destined (No. 1) larvae at Day 3 compared with Day 1–2 (Figure 3a), there were 40 enriched GO terms (Supporting Information Figure S2, Table S16). These included the GO terms related to organ morphogenesis accompanying cell proliferations (e.g., GO:0002009 and 0048729) and those related to carbohydrate metabolisms as well as cuticle development (e.g., GO:0005975, 0035017, and 0042335). There were no GO terms in DEGs between the two periods in worker‐destined (No. 2) larvae (Supporting Information Figure S2).

The characterized GO terms were identified between the No. 1 and No. 2 larvae. Using 465 DEGs at Day 1–2 in soldier‐destined (No. 1) larvae relative to worker‐destined (No. 2) larvae (Figure 3c), 135 enriched GO terms were identified (Supporting Information Figure S2, Table S17). Of the 135 enriched GO terms, there were biological processes related to the cell cycle with chromosome organizations, nuclear division, and organelle fission (e.g., GO:0022402, 0051276, and 0048285). Using 553 DEGs at Day 1–2 in worker‐destined (No. 2) larvae relative to soldier‐destined (No. 1) larvae (Figure 3c), 29 enriched GO terms were identified (Supporting Information Figure S2, Table S18). Of the 29 terms observed in the No. 2 larvae, cuticle development and nutritional metabolism (e.g., GO:0042335 and 1901071) were mainly enriched. These enriched GO terms were also identified in the comparison between the No. 1 and No. 2 larvae at Day 3 using 1,398 and 860 DEGs; a total 221 and 32 GO terms were enriched in the No. 1 and No. 2 larvae, respectively (Supporting Information Figure S2, Tables S19 and S20).

### 3.4 Kyoto Encyclopedia of Genes and Genomes enrichment analysis in differentially expressed genes

Finally, KEGG enrichment analysis (Yu et al., 2012) was performed using DEG data for each gene to reveal the different key metabolic and signaling pathways between soldier‐ and worker‐destined larvae. The results showed enriched KEGG pathways involved in amino acid and sugar metabolism (KEGG ID: dme00350, dme00260, and dme00040) were identified in highly expressed genes in soldier‐destined (No. 1) larvae at Day 1–2 compared with Day 3 (Table 1). However, no pathways were enriched at the two time points in worker‐destined (No. 2) larvae. In the No. 1 larvae, nutritional pathways, such as amino sugar and sucrose metabolism (KEGG ID: dme00520 and dme00500), and an insect hormone biosynthetic pathway (KEGG ID: dme00981), especially ecdysone synthesis genes, were enriched at Day 3 relative to Day 1–2 (Table 1). In the No. 1 larvae compared to the No. 2 larvae, DNA replication (dme03030) and nucleotide synthesis related pathways (e.g., dme03430, dme03420, and dme03410) were upregulated at both Day 1–2 and 3 (Table 1). Moreover, the Hippo signaling pathways (KEGG ID: dme04391 and dme04392) were upregulated at Day 3 (Table 1). On the other hand, in the No. 2 larvae relative to the No. 1 larvae, several metabolic pathways (e.g., KEGG ID: dme00520, dme04142, and dme00350) were enriched at Day 1–2 and Day 3 (Table 1).

**Table 1:** The enriched KEGG pathways in the No. 1 or No. 2 larva

KEGG ID	Description	% in caste‐DEG	% in all	*p *Value	*p*.adjust	*q* Value	Count
(a) Day 1–2 compared with Day 3 in the No. 1 larva							
dme00350	Tyrosine metabolism	10.53	0.68	8.60E−05	3.09E−03	2.26E−03	4
dme00260	Glycine, serine, and threonine metabolism	10.53	1.11	6.41E−04	1.15E−02	8.44E−03	4
dme00040	Pentose and glucuronate interconversions	7.89	0.74	2.32E−03	2.78E−02	2.03E−02	3
(b) Day 3 compared with Day 1–2 in the No. 1 larva							
dme00520	Amino sugar and nucleotide sugar metabolism	13.89	1.84	3.97E−04	1.51E−02	1.29E−02	5
dme00981	Insect hormone biosynthesis	8.33	0.63	1.23E−03	2.33E−02	2.00E−02	3
dme00500	Starch and sucrose metabolism	8.33	0.79	2.44E−03	3.09E−02	2.65E−02	3
(c) The No. 1 larva compared with the No. 2 larva at Day 1–2							
dme03030	DNA replication	23.21	1.32	1.19E−14	4.52E−13	3.25E−13	13
dme00240	Pyrimidine metabolism	16.07	3.00	2.64E−05	3.35E−04	2.41E−04	9
dme03430	Mismatch repair	10.71	0.79	2.05E−06	3.90E−05	2.81E−05	6
dme03420	Nucleotide excision repair	10.71	1.58	1.73E−04	1.64E−03	1.18E−03	6
dme00983	Drug metabolism—other enzymes	8.93	1.32	6.32E−04	4.62E−03	3.33E−03	5
dme03410	Base excision repair	7.14	0.79	7.29E−04	4.62E−03	3.33E−03	4
dme03440	Homologous recombination	5.36	0.74	7.03E−03	3.82E−02	2.75E−02	3
(d) The No. 2 larva compared with the No. 1 larva at Day 1–2							
dme00520	Amino sugar and nucleotide sugar metabolism	18.92	1.84	2.68E−06	8.86E−05	8.20E−05	7
dme04142	Lysosome	16.22	2.79	4.25E−04	7.02E−03	6.49E−03	6
dme00531	Glycosaminoglycan degradation	8.11	0.63	1.33E−03	1.46E−02	1.35E−02	3
(e) The No. 1 larva compared with the No. 2 larva at Day 3							
dme03030	DNA replication	12.87	1.32	3.90E−11	2.26E−09	1.72E−09	13
dme04391	Hippo signaling pathway—fly	9.90	2.11	2.60E−05	7.53E−04	5.74E−04	10
dme03420	Nucleotide excision repair	5.94	1.58	4.09E−03	3.39E−02	2.58E−02	6
dme04392	Hippo signaling pathway—multiple species	4.95	0.68	3.54E−04	6.84E−03	5.21E−03	5
dme03430	Mismatch repair	4.95	0.79	7.58E−04	1.10E−02	8.38E−03	5
dme00511	Other glycan degradation	3.96	0.58	1.87E−03	2.17E−02	1.65E−02	4
dme00531	Glycosaminoglycan degradation	3.96	0.63	2.69E−03	2.60E−02	1.98E−02	4
dme03410	Base excision repair	3.96	0.79	6.55E−03	4.75E−02	3.62E−02	4
(f) The No. 2 larva compared with the No. 1 larva at Day 3							
dme00040	Pentose and glucuronate interconversions	6.90	0.74	3.39E−07	2.27E−05	1.68E−05	8
dme00350	Tyrosine metabolism	5.17	0.68	5.53E−05	1.85E−03	1.37E−03	6

## 4 DISCUSSION

### 4.1 Transcriptomic changes between soldier‐ and worker‐destined larvae

Our results showed that the transcriptome profiles were extraordinarily different between soldier‐destined (No. 1) and worker‐destined (No. 2) larvae, as shown by the MDS plot (Figure 2) and diagrams obtained by ANOVA‐like test (Supporting Information Figure S1). Consequently, there is a possibility that transcriptomic differences might be immediately occurred at early periods (within 3 days) after their 3rd larval molts. The No. 2 larvae always molt into the 4th instar when a particular individual (the No. 1 larva, presoldier or soldier) exists in a colony (Maekawa et al., 2012). Therefore, the transcriptomic differences between the No. 1 and No. 2 larvae are related to the differentiation of each caste (i.e., soldier or worker). There are two previous reports on the transcriptome changes during developments of these larvae, both of which were conducted with no biological replications. The first one indicated that transcriptome profiles were essentially similar among each molting process using only head samples of these larvae and presoldiers (Masuoka, Yaguchi, Toga, Shigenobu, & Maekawa, 2018). The second report showed that a homolog of the lipocalin gene *NLaz* (*ZnNLaz1*) was involved in the soldier differentiation by the regulation of social interaction between the No. 1 larva and a queen (Yaguchi et al., 2018). The present in‐depth study suggests that the different transcriptome profiles at the early periods (within 3 days) during each molt are important for the determination of caste developmental fates. We provide the excellent data to understand the possible genetic networks to integrate social signals and intrinsic factors during soldier differentiation.

Several transcriptome analyses have been performed during caste differentiation in hymenopteran species, all of which suggested that social cues were crucial modulators of gene expression changes (Toth & Rehan, 2017). For example, huge transcriptomic differences were observed among different social contexts in the ants *Pogonomyrmex californicus* and *Cardiocondyla obscurior* (Helmkampf, Mikheyev, Kang, Fewell, & Gadau, 2016; Schrader, Simola, Heinze, & Oettler, 2015). Moreover, the expression levels of nutrition‐sensitive genes involved in caste determination changed in different conditions with/without reproductive castes in the ant *Diacamma* sp. (Okada, Watanabe, Tin, Tsuji, & Mikheyev, 2017). In termites, caste differentiation can occur in response to social signals through inter‐individual interactions, and drastic morphological changes are observed especially during presoldier differentiation (Watanabe et al., 2014). Consequently, there is a possibility that the dynamic transcriptomic differences between Day 1–2 and Day 3 in soldier‐destined (No. 1) larvae observed here (Figure 2) may be triggered by different social interactions. Although the molecular mechanisms underlying social behaviors observed in the No. 1 larvae were poorly understood, neuronal alterations should be required, because the brain levels of biogenic amines (e.g., tyramine and dopamine) are involved in the regulation of social behaviors in termites (Ishikawa, Aonuma, Sasaki, & Miura, 2016; Yaguchi et al., 2016). We suggest that transcriptomic differences between the No. 1 and No. 2 larvae are attributed not only to physiological changes for soldier‐specific traits but also to behavioral changes in a context‐dependent environment.

### 4.2 The possible transcriptomic changes in response to the nutritional differences

In social insects, nutritional signals are involved in caste‐specific developments and behaviors through endocrine regulations (Smith, Toth, Suarez, & Robinson, 2008). The nutritional differences were observed between the soldier and worker developmental processes in an incipient colony of *Z. nevadensis* (Maekawa et al., 2012). The *ZnNLaz1* was crucial factor for soldier differentiation through the regulation of trophallactic interactions with a queen (Yaguchi et al., 2018). In *D. melanogaster*, *NLaz* was assigned as one of the GO terms such as lipid metabolic process (GO:0006629). When *NLaz* was overexpressed in flies, lipid contents were significantly changed in adult individuals (Hull‐Thompson et al., 2009). In the present study, lipid metabolic and biosynthetic processes (GO:0006629 and 0008610) were identified in the No. 1 larvae at Day 1–2 compared with Day 3 (Supporting Information Table S15), but *ZnNLaz1 *(Znev_05665) was highly expressed at Day 3 (Supporting Information Tables S4 and S9), as shown in the previous work (Yaguchi et al., 2018). Although the certain relationships between *ZnNLaz1 *and lipid contents are unknown in termites, there is a possibility that the nutritional contents (e.g., lipid and related compounds) are frequently received from a queen by the regulation of *ZnNLaz1* expression. Alternatively, larval nutritional conditions may be enhanced by the highly *ZnNLaz1* expression during the soldier developments. Further physiological cross talk between nutritional status and *ZnNLaz1 *expression should be analyzed to clarify the role of endocrine regulations during soldier differentiation.

Compared to soldier‐destined (No. 1) larvae, GO terms involved in metabolic process were mainly upregulated in worker‐destined (No. 2) larvae. No GO terms were enriched in the No. 2 larvae between Day 1–2 and Day 3, but 4 terms (GO:0006022, 0006030, 0006040, and 1901071) were commonly upregulated in the No. 2 larvae compared to the No. 1 larvae at both time points (Supporting Information Tables S18 and S20). There is a possibility that these metabolic pathways are related to the response to nutritional intakes in the No. 2 larvae. Interestingly, these four GO terms were also upregulated in the worker‐biased genes in 16 ant species, even though these terms were not related to worker sterility (Morandin et al., 2016). These four GO terms were also enriched within nourishment‐responsive genes in the paper wasp *Polistes metricus *(Berens, Hunt, & Toth, 2015). In addition, the pentose‐related pathway (dme00040) was also upregulated in the No. 2 larvae at Day 3 (Table 1). This metabolic pathway was identified in the caste‐biased genes in the ant *Diacamma* sp. (Okada et al., 2017). Consequently, these metabolic and nutritional pathways may be potentially conserved modules (“genetic toolkits”; Toth & Rehan, 2017) also in termites. Further analyses should be performed to know how these pathways are involved in the termite worker formation through nutritional intakes.

### 4.3 The potential molecular mechanisms of soldier differentiation

We identified potential genetic components underlying soldier differentiation. First, a KEGG pathway related to insect hormone biosynthesis (dme00981) was identified at Day 3 compared with Day 1–2 in soldier‐destined (No. 1) larvae (Table 1). Especially, expression levels of three genes involved in ecdysone synthesis (*shadow*, *shade,* and *spookier*) were highly upregulated. This was consistent with the previous reports, which showed that ecdysone receptor signaling activity was important for presoldier and soldier formations in *Z. nevadensis* (Masuoka & Maekawa, 2016), and that the caste‐specific expressions of ecdysone synthesis genes were broadly observed among three termite species including *Z. nevadensis* (Harrison et al., 2018).

Second, in the No. 1 larva relative to the No. 2 larva, GO terms involved in the cell cycle (e.g., GO:0000278 and 0022402) were upregulated (Supporting Information Tables S17 and S19). Outer cuticles, especially in the head and mandibles, developed rapidly after the gut‐purged period of the No. 1 larvae (Masuoka et al., 2015), and the total body sizes were quite different between presoldiers and the 4th‐instar larvae (Itano & Maekawa, 2008). These morphological changes may be regulated by genes involved in the cell cycle, chromosome organization, and tissue morphogenesis (e.g., GO:0000278, 0022402, 0051276, and 0048729). In the migratory locust *Locusta migratoria*, minichromosome maintenance (MCM) genes in DNA replication pathway were positively involved in the DNA synthesis of fat body cells through JH actions (Guo et al., 2014). These KEGG pathways including MCM genes were enriched in the No. 1 larvae relative to the No. 2 larvae at both time points (Table 1). During termite soldier differentiation, cell numbers and sizes in the fat body were increased in *Hodotermopsis sjostedti* (Cornette, Matsumoto, & Miura, 2007), and JH biosynthetic genes were highly expressed in *Z. nevadensis* (Yaguchi et al., 2015). Thus, it is possible that the genes involved in DNA replication and cell proliferation may be responsible for soldier development through JH actions.

Third, KEGG enrichment analysis showed that the Hippo signaling pathway (dme04391 and dme04392) were significantly upregulated in the No. 1 larvae at Day 3. The Hippo signaling pathway is broadly conserved among animals including mammals and insects (Yu & Guan, 2013). This pathway comprises a serine–threonine kinase cascade and mediates organ and tissue growth through the regulation of cell proliferation (Pan, 2010). For example, a mutant of *warts*, one of the members of this pathway, resulted in tumorous overgrowth of Notum in *D. melanogaster* adults (Staley & Irvine, 2012), and a transcriptional coactivator Yorkie, the downstream target of warts, was involved in tissue growth of *D. melanogaster* and the silkworm *Bombyx mori* (Huang, Wu, Barrera, Matthews, & Pan, 2005; Xu et al., 2018). In the rhinoceros beetle *Trypoxylus dichotomus*, knockdown of *ds* and *ft* in this pathway resulted in a decrease in head and thoracic horn size (Hust et al., 2018). Although the roles of Hippo signaling pathway in insects other than the holometabola (with a pupal stage) are still unknown, the present results suggest that soldier‐specific exaggerated morphogenesis (e.g., mandibular elongation and head enlargement) is also regulated by this pathway in response to high nutritional conditions. The Hippo signaling pathway might play an important potential role in the integration of nutritional signals and endocrine factors, which resulted in specific organ morphogenesis in some beetles (Casasa, Schwab, & Moczek, 2017; Gotoh et al., 2015). Given that Hippo signaling pathway is broadly conserved among animals (Yu & Guan, 2013), they may also be crucial for termite soldier differentiation via the integration of nutrition and hormone signals. Because food intake from reproductives was frequently observed in the No. 1 larvae at Day 3 after their appearance (Maekawa et al., 2012), nutritional conditions may enhance the growth of these larval bodies. Moreover, recent work suggested that the presoldier molts were positively regulated by the TGF‐beta signaling pathway through hormonal actions in *Z. nevadensis* (Masuoka et al., 2018). There is a possible cross talk between TGF and Hippo signaling pathways in *D. melanogaster* (Pan, 2010). Further expression and function analysis of components in both pathways are needed to clarify a possible role for soldier developments through nutritional intakes.

## 5 CONCLUSIONS

The complex social organization of termites can be achieved by social interactions among multiple phenotypes (i.e., castes). Importantly, social interactions contributed to the determination of larval developmental fates (Watanabe et al., 2014). Here, we could identify clear lists of DEGs between the soldier‐ and worker‐destined individuals by focusing on larval development in an incipient colony of *Z. nevadensis*. The present findings suggest that huge transcriptomic changes are involved in the regulation of caste differentiation. The obtained lists of DEGs represent a cornerstone for our understanding of how termite individuals are determined for the development of each caste under the influence of the social signals through inter‐individual interactions.

## CONFLICT OF INTEREST

None declared.

## AUTHORS’ CONTRIBUTIONS

H.Y., R.S., and K.M. designed the study; H.Y., R.S., and K.M. collected samples; H.Y., R.S., and M.M. performed the experiments; H.Y., R.S., M.M., and S.S. analyzed the data; H.Y., R.S., and K.M. drafted the manuscript; all authors contributed to the final version of the manuscript.

## Data Availability

RNA‐seq data obtained are available from the DDBJ Sequence Read Archive (DRA) database (accession no. DRA007363). All other relevant data are within the paper and its electronic supplementary material.

## References

[ece34976-bib-0001] Andrews, S. (2010). FastQC: A quality control tool for high throughput sequence data. Retrieved from http://www.bioinformatics.babraham.ac.uk/projects/fastqc

[ece34976-bib-0002] Benjamini, Y. , & Hochberg, Y. (1995). Controlling the false discovery rate: A practical and powerful approach to multiple testing. Journal of the Royal Statistical Society B, 57, 289–300.

[ece34976-bib-0003] Berens, A. J. , Hunt, J. H. , & Toth, A. L. (2015). Nourishment level affects caste‐related gene expression in *Polistes wasps* . BMC Genomics, 16, 235. 25880983 10.1186/s12864-015-1410-y PMC4381360

[ece34976-bib-0004] Bordereau, C. , & Han, S. H. (1986). Stimulatory influence of the queen and king on soldier differentiation in the higher termites *Nasutitermes lujae* and *Cubitermes fungifaber* . Insectes Sociaux, 33, 296–305.

[ece34976-bib-0005] Carlson, M. R. J. , Pagès, H. , Arora, S. , Obenchain, V. , & Morgan, M. (2016). Genomic annotation resources in R/Bioconductor. Methods in Molecular Biology, 1418, 67–90. 27008010 10.1007/978-1-4939-3578-9_4

[ece34976-bib-0006] Casasa, S. , Schwab, D. B. , & Moczek, A. M. (2017). Developmental regulation and evolution of scaling: Novel insights through the study of *Onthophagus* beetles. Current Opinion in Insect Science, 19, 52–60. 28521943 10.1016/j.cois.2016.11.004

[ece34976-bib-0007] Cornette, R. , Matsumoto, T. , & Miura, T. (2007). Histological analysis of fat body development and molting events during soldier differentiation in the damp‐wood termite, *Hodotermopsis sjostedti* (Isoptera, Termopsidae). Zoological Science, 24, 1066–1074. 10.2108/zsj.24.1066 18348606

[ece34976-bib-0008] Cox, M. P. , Peterson, D. A. , & Biggs, P. J. (2010). SolexaQA: At‐a‐glance quality assessment of Illumina second‐generation sequencing data. BMC Bioinformatics, 11, 485 10.1186/1471-2105-11-485 20875133 PMC2956736

[ece34976-bib-0009] Gilbert, S. F. , & Epel, D. (2009). Ecological developmental biology: Integrating epigenetics, medicine, and evolution. Sunderland, MA: Sinauer Associates Inc.

[ece34976-bib-0010] Glastad, K. M. , Gokhale, K. , Liebig, J. , & Goodisman, M. A. (2016). The caste‐ and sex‐specific DNA methylome of the termite *Zootermopsis nevadensis* . Scientific Reports, 6, 37110. 27848993 10.1038/srep37110 PMC5111047

[ece34976-bib-0011] Gotoh, H. , Hust, J. A. , Miura, T. , Niimi, T. , Emlen, D. J. , & Lavine, L. C. (2015). The Fat/Hippo signaling pathway links within‐disc morphogen patterning to whole‐animal signals during phenotypically plastic growth in insects. Developmental Dynamics, 244, 1039–1045. 25997872 10.1002/dvdy.24296

[ece34976-bib-0012] Guo, W. , Wu, Z. , Song, J. , Jiang, F. , Wang, Z. , Deng, S. , … Zhou, S. (2014). Juvenile hormone‐receptor complex acts on *Mcm4* and *Mcm7* to promote polyploidy and vitellogenesis in the migratory locust. PLoS Genetics, 10, e1004702. 25340846 10.1371/journal.pgen.1004702 PMC4207617

[ece34976-bib-0013] Harrison, M. C. , Jongepier, E. , Robertson, H. M. , Arning, N. , Bitard‐Feildel, T. , Chao, H. , … Bornberg‐Bauer, E. (2018). Hemimetabolous genomes reveal molecular basis of termite eusociality. Nature Ecology & Evolution, 2, 557–566. 29403074 10.1038/s41559-017-0459-1 PMC6482461

[ece34976-bib-0014] Helmkampf, M. , Mikheyev, A. S. , Kang, Y. , Fewell, J. , & Gadau, J. (2016). Gene expression and variation in social aggression by queens of the harvester ant *Pogonomyrmex californicus* . Molecular Ecology, 25, 3716–3730. 27178446 10.1111/mec.13700

[ece34976-bib-0015] Huang, J. , Wu, S. , Barrera, J. , Matthews, K. , & Pan, D. (2005). The Hippo signaling pathway coordinately regulates cell proliferation and apoptosis by inactivating Yorkie, the *Drosophila* homolog of YAP. Cell, 122, 421–434. 10.1016/j.cell.2005.06.007 16096061

[ece34976-bib-0016] Hull‐Thompson, J. , Muffat, J. , Sanchez, D. , Walker, D. W. , Benzer, S. , Ganfornina, M. D. , & Jasper, H. (2009). Control of metabolic homeostasis by stress signaling is mediated by the lipocalin NLaz. PLoS Genetics, 5, e1000460. 19390610 10.1371/journal.pgen.1000460 PMC2667264

[ece34976-bib-0017] Hust, J. , Lavine, M. D. , Worthington, A. M. , Zinna, R. , Gotoh, H. , Niimi, T. , & Lavine, L. (2018). The Fat‐Dachsous signaling pathway regulates growth of horns in *Trypoxylus dichotomus*, but does not affect horn allometry. Journal of Insect Physiology, 105, 85–94. 10.1016/j.jinsphys.2018.01.006 29366850

[ece34976-bib-0018] Ishikawa, Y. , Aonuma, H. , Sasaki, K. , & Miura, T. (2016). Tyraminergic and octopaminergic modulation of defensive behavior in termite soldier. PLoS ONE, 11, e0154230. 27196303 10.1371/journal.pone.0154230 PMC4873212

[ece34976-bib-0019] Itano, H. , & Maekawa, K. (2008). Soldier differentiation and larval juvenile hormone sensitivity in an incipient colony of the damp‐wood termite *Zootermopsis nevadensis* (Isoptera, Termopsidae). Sociobiology, 51, 151–162.

[ece34976-bib-0020] Kim, D. , Pertea, G. , Trapnell, C. , Pimentel, H. , Kelley, R. , & Salzberg, S. L. (2013). TopHat2: Accurate alignment of transcriptomes in the presence of insertions, deletions and gene fusions. Genome Biology, 14, R36 10.1186/gb-2013-14-4-r36 23618408 PMC4053844

[ece34976-bib-0021] Liao, Y. , Smyth, G. K. , & Shi, W. (2014). featureCounts: An efficient general purpose program for assigning sequence reads to genomic features. Bioinformatics, 30, 923–930. 10.1093/bioinformatics/btt656 24227677

[ece34976-bib-0022] Linksvayer, T. A. , Fewell, J. H. , Gadau, J. , & Laubichler, M. D. (2012). Developmental evolution in social insects: Regulatory networks from genes to societies. Journal of Experimental Zoology Part B Molecular and Developmental Evolution, 318, 159–169. 10.1002/jez.b.22001 22544713

[ece34976-bib-0023] Maekawa, K. , Nakamura, S. , & Watanabe, D. (2012). Termite soldier differentiation in incipient colonies is related to parental proctodeal trophallactic behavior. Zoological Science, 29, 213–217. 22468829 10.2108/zsj.29.213

[ece34976-bib-0024] Martin, M. (2011). Cutadapt removes adapter sequences from high‐throughput sequencing reads. EMBnet.journal, 17, 10–12.

[ece34976-bib-0025] Masuoka, Y. , & Maekawa, K. (2016). Ecdysone signaling regulates soldier‐specific cuticular pigmentation in the termite *Zootermopsis nevadensis* . FEBS Letters, 590, 1694–1703. 27208413 10.1002/1873-3468.12219

[ece34976-bib-0026] Masuoka, Y. , Yaguchi, H. , Suzuki, R. , & Maekawa, K. (2015). Knockdown of the juvenile hormone receptor gene inhibits soldier‐specific morphogenesis in the damp‐wood termite *Zootermopsis nevadensis* (Isoptera: Archotermopsidae). Insect Biochemistry and Molecular Biology, 64, 25–31. 26188329 10.1016/j.ibmb.2015.07.013

[ece34976-bib-0027] Masuoka, Y. , Yaguchi, H. , Toga, K. , Shigenobu, S. , & Maekawa, K. (2018). TGF*β* signaling related genes are involved in hormonal mediation during termite soldier differentiation. PLoS Genetics, 14, e1007338. 29641521 10.1371/journal.pgen.1007338 PMC5912798

[ece34976-bib-0028] McCarthy, D. J. , Chen, Y. , & Smyth, G. K. (2012). Differential expression analysis of multifactor RNA‐Seq experiments with respect to biological variation. Nucleic Acids Research, 40, 4288–4297. 22287627 10.1093/nar/gks042 PMC3378882

[ece34976-bib-0029] Mitaka, Y. , Mori, N. , & Matsuura, K. (2017). Multi‐functional roles of a soldier‐specific volatile as a worker arrestant, primer pheromone and an antimicrobial agent in a termite. Proceedings of the Royal Society B. Biological Sciences, 284, 20171134. 10.1098/rspb.2017.1134 PMC5543234 28747483

[ece34976-bib-0030] Miura, T. , & Scharf, M. E. (2011). Molecular basis underlying caste differentiation in termites In Bignell D. E., Roisin Y., & Lo N. (Eds.), Biology of termites: A modern synthesis (pp. 211–253). Heidelberg, Germany: Springer.

[ece34976-bib-0031] Morandin, C. , Tin, M. M. Y. , Abril, S. , Gómez, C. , Pontieri, L. , Schiøtt, M. , … Mikheyev, A. S. (2016). Comparative transcriptomics reveals the conserved building blocks involved in parallel evolution of diverse phenotypic traits in ants. Genome Biology, 17, 43. 26951146 10.1186/s13059-016-0902-7 PMC4780134

[ece34976-bib-0032] Nalepa, C. A. (2011). Altricial development in wood‐feeding cockroaches: The key antecedent of termite eusociality In Bignell D. E., Roisin Y., & Lo N. (Eds.), Biology of termites: A modern synthesis (pp. 69–95). Heidelberg, Germany: Springer.

[ece34976-bib-0033] Noirot, C. (1991). Caste differentiation in Isoptera: Basic features, role of pheromones. Ethology Ecology & Evolution, Special Issue, 1, 3–7.

[ece34976-bib-0034] Okada, Y. , Watanabe, Y. , Tin, M. M. Y. , Tsuji, K. , & Mikheyev, A. S. (2017). Social dominance alters nutrition‐related gene expression immediately: Transcriptomic evidence from a monomorphic queenless ant. Molecular Ecology, 26, 2922–2938. 10.1111/mec.13989 28036149

[ece34976-bib-0035] Pan, D. (2010). The hippo signaling pathway in development and cancer. Developmental Cell, 19, 491–505. 20951342 10.1016/j.devcel.2010.09.011 PMC3124840

[ece34976-bib-0036] R Core Team (2017). R: A language and environment for statistical computing. Vienna, Austria: R Foundation for Statistical Computing Retrieved from https://www.R-project.org/

[ece34976-bib-0037] Robinson, M. D. , McCarthy, D. J. , & Smyth, G. K. (2010). edgeR: A Bioconductor package for differential expression analysis of digital gene expression data. Bioinformatics, 26, 139–140. 10.1093/bioinformatics/btp616 19910308 PMC2796818

[ece34976-bib-0038] Robinson, M. D. , & Oshlack, A. (2010). A scaling normalization method for differential expression analysis of RNA‐seq data. Genome Biology, 11, R25. 20196867 10.1186/gb-2010-11-3-r25 PMC2864565

[ece34976-bib-0039] Schrader, L. , Simola, D. F. , Heinze, J. , & Oettler, J. (2015). Sphingolipids, transcription factors, and conserved toolkit genes: Developmental plasticity in the ant *Cardiocondyla obscurior* . Molecular Biology and Evolution, 32, 1474–1486. 25725431 10.1093/molbev/msv039 PMC4615751

[ece34976-bib-0040] Simpson, S. J. , Sword, G. A. , & Lo, N. (2011). Polyphenism in insects. Current Biology, 21, R738–R749. 21959164 10.1016/j.cub.2011.06.006

[ece34976-bib-0041] Smith, C. R. , Toth, A. L. , Suarez, A. V. , & Robinson, G. E. (2008). Genetic and genomic analyses of the division of labour in insect societies. Nature Review Genetics, 9, 735–748. 10.1038/nrg2429 18802413

[ece34976-bib-0042] Staley, B. K. , & Irvine, K. D. (2012). Hippo signaling in *Drosophila*: Recent advances and insights. Developmental Dynamics, 241, 3–15. 22174083 10.1002/dvdy.22723 PMC3426292

[ece34976-bib-0043] Terrapon, N. , Li, C. , Robertson, H. M. , Ji, L. , Meng, X. , Booth, W. , … Liebig, J. (2014). Molecular traces of alternative social organization in a termite genome. Nature Communications, 5, 3636. 10.1038/ncomms4636 24845553

[ece34976-bib-0044] Tian, L. , & Zhou, X. (2014). The soldiers in societies: Defense, regulation, and evolution. International Journal of Biological Sciences, 10, 296–308. 24644427 10.7150/ijbs.6847 PMC3957085

[ece34976-bib-0045] Toth, A. L. , & Rehan, S. M. (2017). Molecular evolution of insect sociality: An eco‐evo‐devo perspective. Annual Review of Entomology, 62, 419–442. 10.1146/annurev-ento-031616-035601 27912247

[ece34976-bib-0046] Watanabe, D. , Gotoh, H. , Miura, T. , & Maekawa, K. (2011). Soldier presence suppresses presoldier differentiation through a rapid decrease of JH in the termite *Reticulitermes speratus* . Journal of Insect Physiology, 57, 791–795. 21414320 10.1016/j.jinsphys.2011.03.005

[ece34976-bib-0047] Watanabe, D. , Gotoh, H. , Miura, T. , & Maekawa, K. (2014). Social interaction affecting caste development through physiological actions in termites. Frontiers in Physiology, 5, 1–12. 24782780 10.3389/fphys.2014.00127 PMC3988372

[ece34976-bib-0048] Weesner, F. M. (1969). External anatomy In Krishna K., & Weesner F. M. (Eds.), Biology of termites (vol. I, pp. 19–47). New York, NY: Academic Press.

[ece34976-bib-0049] Wilson, E. O. (1971). The insect societies. Cambridge, UK: Harvard University Press.

[ece34976-bib-0050] Xu, X. , Zhang, Z. , Yang, Y. , Huang, S. , Li, K. , He, L. , & Zhou, L. (2018). Genome editing reveals the function of Yorkie during the embryonic and early larval development in silkworm, *Bombyx mori* . Insect Molecular Biology, 27, 675–685. 29797485 10.1111/imb.12502

[ece34976-bib-0051] Yaguchi, H. , Inoue, T. , Sasaki, K. , & Maekawa, K. (2016). Dopamine regulates termite soldier differentiation through trophallactic behaviours. Royal Society Open Science, 3, 150574. 26998327 10.1098/rsos.150574 PMC4785978

[ece34976-bib-0052] Yaguchi, H. , Masuoka, Y. , Inoue, T. , & Maekawa, K. (2015). Expressions of juvenile hormone biosynthetic genes during presoldier differentiation in the incipient colony of *Zootermopsis nevadensis* (Isoptera: Archotermopsidae). Applied Entomology and Zoology, 50, 497–508. 10.1007/s13355-015-0358-3

[ece34976-bib-0053] Yaguchi, H. , Shigenobu, S. , Hayashi, Y. , Miyazaki, S. , Toga, K. , Masuoka, Y. , & Maekawa, K. (2018). A lipocalin protein, Neural Lazarillo, is key to social interactions that promote termite soldier differentiation. Proceedings of the Royal Society B. Biological Sciences, 285, 20180707. 10.1098/rspb.2018.0707 PMC6083259 30051867

[ece34976-bib-0054] Yu, F.‐X. , & Guan, K.‐L. (2013). The hippo pathway: Regulators and regulations. Genes & Development, 27, 355–371. 23431053 10.1101/gad.210773.112 PMC3589553

[ece34976-bib-0055] Yu, G. , Wang, L.‐G. , Han, Y. , & He, Q.‐Y. (2012). clusterProfiler: An R package for comparing biological themes among gene clusters. OMICS: A Journal of Integrative Biology, 16, 284–287. 22455463 10.1089/omi.2011.0118 PMC3339379

