# Supplementary figures and images for: Transcriptomic changes during caste development through social interactions in the termite Zootermopsis nevadensis
Source: Ecol Evol. 2019 Feb 23;9(6):3446–56. doi: 10.1002/ece3.4976 (PMC6434549; doi:10.1002/ece3.4976)

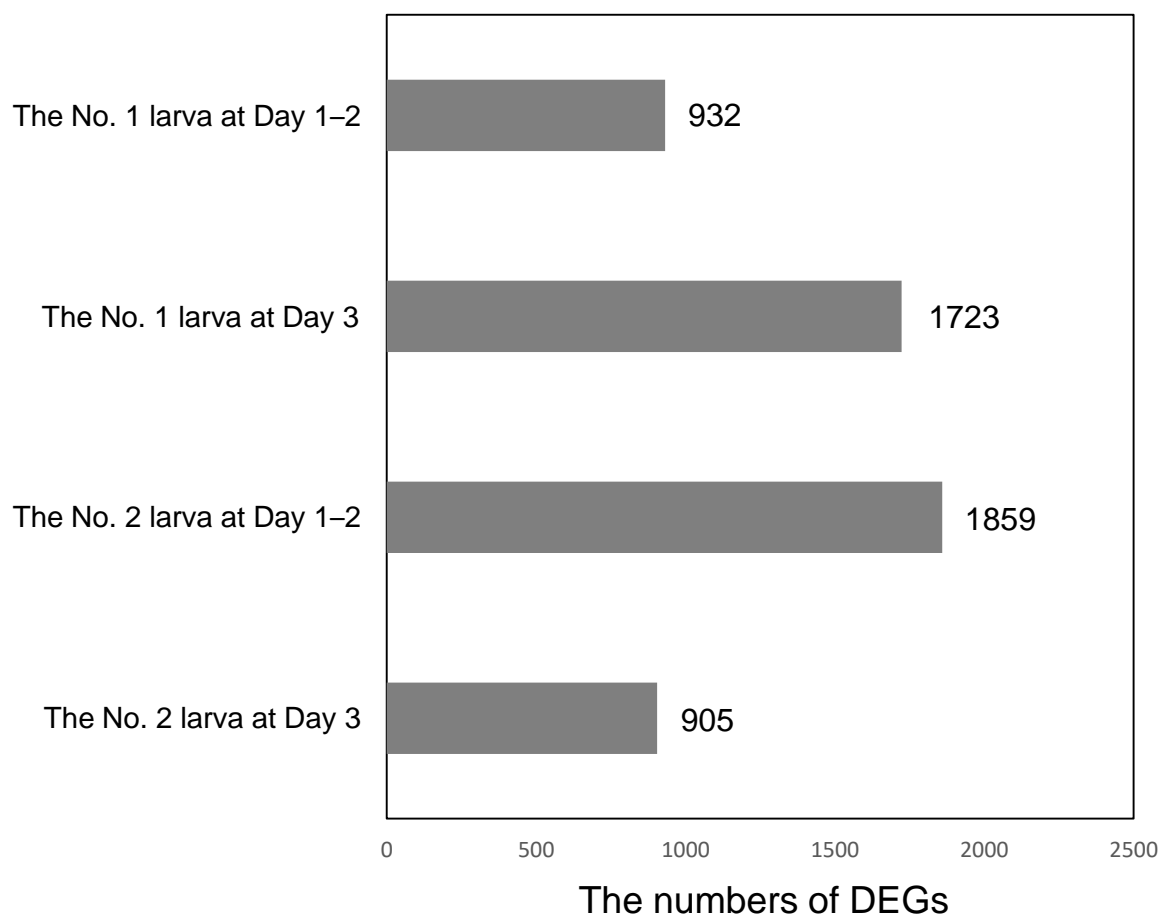

Fig. S1

Supplement: Supplementary file 1 [file ECE3-9-3446-s001.pdf]

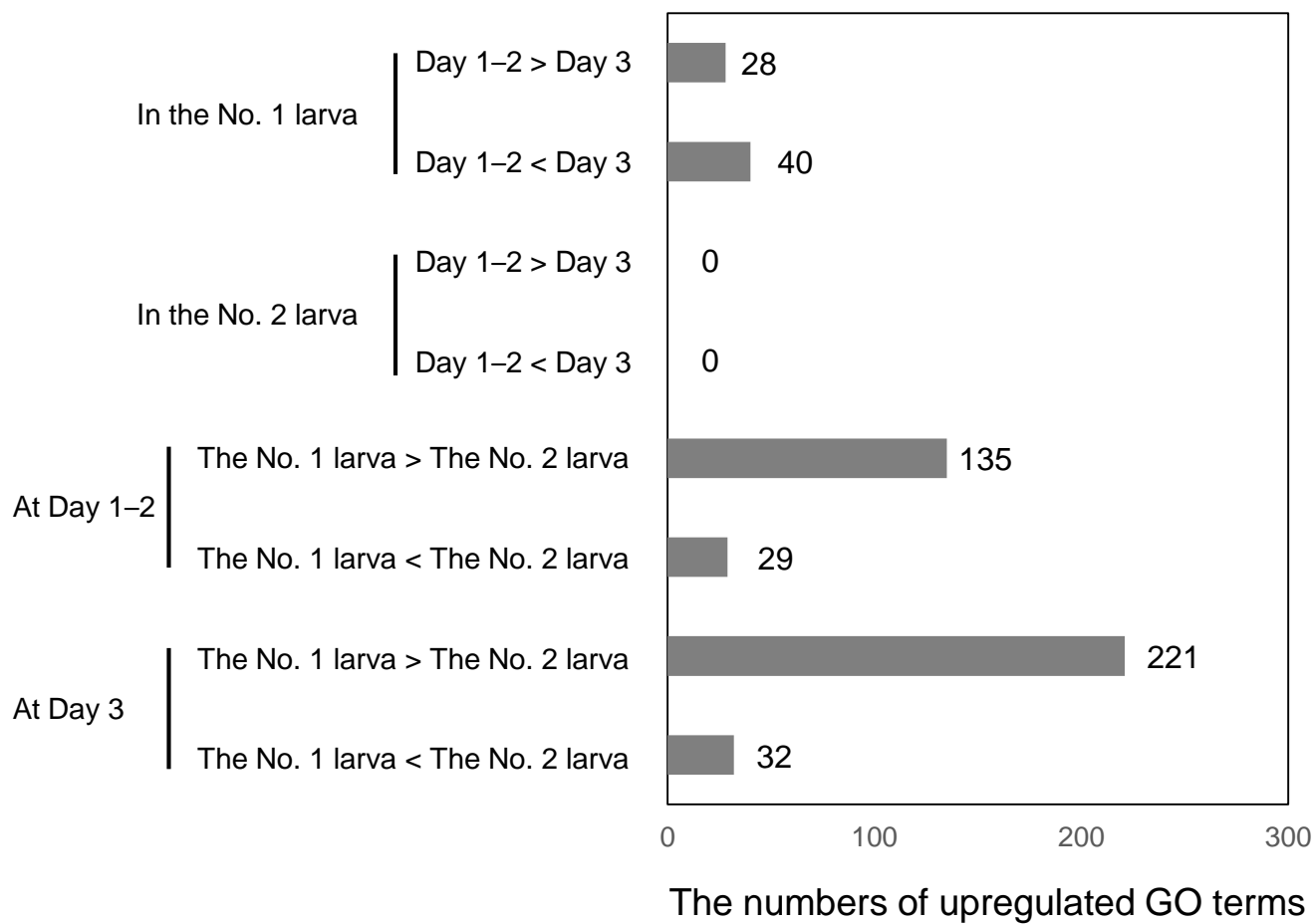

Fig. S2

Supplement: Supplementary file 2 [file ECE3-9-3446-s002.pdf]
