# Supplementary material for: Transcriptomic changes during caste development through social interactions in the termite Zootermopsis nevadensis
Source: Ecol Evol. 2019 Feb 23;9(6):3446–56. doi: 10.1002/ece3.4976 (PMC6434549; doi:10.1002/ece3.4976)
Supplement: Supplementary file 3 [file ECE3-9-3446-s003.pdf]

Table S1. The used incipient colonies for RNA-seq analyses.

Incipient colony No.	Collection larva	Collection day	Presoldier present or absent*	RNA-seq library No.
Incipient colony 1	No. 1 larva	Day 1	absent	1
Incipient colony 2	No. 1 larva	Day 1	absent	1
Incipient colony 3	No. 1 larva	Day 1	absent	2
Incipient colony 4	No. 1 larva	Day 1	absent	2
Incipient colony 5	No. 1 larva	Day 1	absent	3
Incipient colony 6	No. 1 larva	Day 1	absent	3
Incipient colony 7	No. 1 larva	Day 2	absent	1
Incipient colony 8	No. 1 larva	Day 2	absent	1
Incipient colony 9	No. 1 larva	Day 2	absent	2
Incipient colony 10	No. 1 larva	Day 2	absent	2
Incipient colony 11	No. 1 larva	Day 2	absent	3
Incipient colony 12	No. 1 larva	Day 2	absent	3
Incipient colony 13	No. 1 larva	Day 3	absent	4
Incipient colony 14	No. 1 larva	Day 3	absent	4
Incipient colony 15	No. 1 larva	Day 3	absent	4
Incipient colony 16	No. 1 larva	Day 3	absent	4
Incipient colony 17	No. 1 larva	Day 3	absent	5
Incipient colony 18	No. 1 larva	Day 3	absent	5
Incipient colony 19	No. 1 larva	Day 3	absent	5
Incipient colony 20	No. 1 larva	Day 3	absent	5
Incipient colony 21	No. 1 larva	Day 3	absent	6
Incipient colony 22	No. 1 larva	Day 3	absent	6
Incipient colony 23	No. 1 larva	Day 3	absent	6
Incipient colony 24	No. 1 larva	Day 3	absent	6
Incipient colony 25	No. 2 larva	Day 1	present	7
Incipient colony 26	No. 2 larva	Day 1	present	7
Incipient colony 27	No. 2 larva	Day 1	present	8
Incipient colony 28	No. 2 larva	Day 1	present	8
Incipient colony 29	No. 2 larva	Day 1	present	9
Incipient colony 30	No. 2 larva	Day 1	present	9
Incipient colony 31	No. 2 larva	Day 2	present	7
Incipient colony 32	No. 2 larva	Day 2	present	7
Incipient colony 33	No. 2 larva	Day 2	present	8
Incipient colony 34	No. 2 larva	Day 2	present	8
Incipient colony 35	No. 2 larva	Day 2	present	9

Incipient colony 36	No. 2 larva	Day 2	present	9
Incipient colony 37	No. 2 larva	Day 3	present	10
Incipient colony 38	No. 2 larva	Day 3	present	10
Incipient colony 39	No. 2 larva	Day 3	present	10
Incipient colony 40	No. 2 larva	Day 3	present	10
Incipient colony 41	No. 2 larva	Day 3	present	11
Incipient colony 42	No. 2 larva	Day 3	present	11
Incipient colony 43	No. 2 larva	Day 3	present	11
Incipient colony 44	No. 2 larva	Day 3	present	11
Incipient colony 45	No. 2 larva	Day 3	present	12
Incipient colony 46	No. 2 larva	Day 3	present	12
Incipient colony 47	No. 2 larva	Day 3	present	12
Incipient colony 48	No. 2 larva	Day 3	present	12

* Presoldier is differented from the No. 1 larva
