# Supplementary material for: Transcriptomic changes during caste development through social interactions in the termite Zootermopsis nevadensis
Source: Ecol Evol. 2019 Feb 23;9(6):3446–56. doi: 10.1002/ece3.4976 (PMC6434549; doi:10.1002/ece3.4976)
Supplement: Supplementary file 4 [file ECE3-9-3446-s004.pdf]

Table S2. The RNA-seq library information.

	Raw reads	The used reads	Mapping rate (%)
No. 1; Day 1 - 2; replication 1	13242716	12689307	76.4
No. 1; Day 1 - 2; replication 2	11429761	10919652	79.7
No. 1; Day 1 - 2; replication 3	12006046	11445602	75.3
No. 1; Day 3; replication 1	11509269	11108981	79.9
No. 1; Day 3; replication 2	11930734	11451207	79.1
No. 1; Day 3; replication 3	12127919	11668616	79.1
No. 2; Day 1 - 2; replication 1	13316987	12844092	75.4
No. 2; Day 1 - 2; replication 2	11536090	11115439	76.5
No. 2; Day 1 - 2; replication 3	10727018	10162122	76.7
No. 2; Day 3; replication 1	11036350	10656778	75.2
No. 2; Day 3; replication 2	10132628	9760330	72.8
No. 2; Day 3; replication 3	9860251	9411526	71.3
