# Supplementary material for: Transcriptomic changes during caste development through social interactions in the termite Zootermopsis nevadensis
Source: Ecol Evol. 2019 Feb 23;9(6):3446–56. doi: 10.1002/ece3.4976 (PMC6434549; doi:10.1002/ece3.4976)
Supplement: Supplementary file 7 [file ECE3-9-3446-s007.pdf]

Table S5. The upregulated genes at Day 1–2 compared with Day 3 in the No. 2 larva.

Gene ID	logFC	logCPM	LR	PValue	FDR
Znev_00645	-2.845658197	2.539982171	43.75910223	3.71E-11	5.90E-07
Znev_06900	-1.245263098	4.36963924	24.31135304	8.20E-07	2.60E-03
Znev_07832	-0.949722169	6.304185011	23.55806432	1.21E-06	3.21E-03
