# Supplementary material for: Transcriptomic changes during caste development through social interactions in the termite Zootermopsis nevadensis
Source: Ecol Evol. 2019 Feb 23;9(6):3446–56. doi: 10.1002/ece3.4976 (PMC6434549; doi:10.1002/ece3.4976)
Supplement: Supplementary file 8 [file ECE3-9-3446-s008.pdf]

Table S6. The upregulated genes at Day 3 compared with Day 1–2 in the No. 2 larva.

Gene ID	logFC	logCPM	LR	PValue	FDR
Znev_04285	0.987790507	5.212205659	23.01809705	1.60E-06	3.64E-03
Znev_02966	1.297218222	6.736698045	29.06902685	6.98E-08	3.70E-04
Znev_13261	1.538074468	3.094771269	18.7629271	1.48E-05	2.35E-02
Znev_02536	1.579691572	2.113850072	21.25745249	4.02E-06	7.08E-03
Znev_05498	1.951938619	7.144762659	31.40795383	2.09E-08	1.66E-04
Znev_02607	2.444725559	2.99128867	26.4047	2.77E-07	1.10E-03
Znev_04323	3.40985056	0.492191253	21.85119854	2.95E-06	5.85E-03
