# Supplementary material for: Transcriptomic changes during caste development through social interactions in the termite Zootermopsis nevadensis
Source: Ecol Evol. 2019 Feb 23;9(6):3446–56. doi: 10.1002/ece3.4976 (PMC6434549; doi:10.1002/ece3.4976)
Supplement: Supplementary file 17 [file ECE3-9-3446-s017.pdf]

Table S15. The enriched GO terms at Day 1-2 compared with Day 3 in the No. 1 larva.

ID	Description	% in caste-DEG	% in all	pvalue	p.adjust	qvalue	Count
GO:0055114	oxidation-reduction process	19.79	6.25	5.17E-06	1.26E-03	1.19E-03	19
GO:0019752	carboxylic acid metabolic process	15.63	4.78	4.09E-05	5.11E-03	4.84E-03	15
GO:0006082	organic acid metabolic process	15.63	5.17	1.00E-04	8.05E-03	7.63E-03	15
GO:0043436	oxoacid metabolic process	15.63	5.17	1.00E-04	8.05E-03	7.63E-03	15
GO:0006629	lipid metabolic process	14.58	5.33	4.94E-04	2.18E-02	2.06E-02	14
GO:0008610	lipid biosynthetic process	10.42	2.36	7.47E-05	8.01E-03	7.58E-03	10
GO:0042335	cuticle development	9.38	2.22	2.43E-04	1.21E-02	1.15E-02	9
GO:0044283	small molecule biosynthetic process	8.33	2.18	1.06E-03	3.18E-02	3.01E-02	8
GO:0006030	chitin metabolic process	7.29	0.59	9.60E-07	7.20E-04	6.82E-04	7
GO:1901071	glucosamine-containing compound metabolic process	7.29	0.71	3.76E-06	1.26E-03	1.19E-03	7
GO:0006040	amino sugar metabolic process	7.29	0.77	6.72E-06	1.26E-03	1.19E-03	7
GO:0006022	aminoglycan metabolic process	7.29	0.96	2.90E-05	4.35E-03	4.12E-03	7
GO:0032787	monocarboxylic acid metabolic process	7.29	1.77	1.43E-03	3.82E-02	3.62E-02	7
GO:0055088	lipid homeostasis	6.25	0.86	1.42E-04	8.85E-03	8.38E-03	6
GO:0006631	fatty acid metabolic process	6.25	1.12	6.38E-04	2.52E-02	2.38E-02	6
GO:0016053	organic acid biosynthetic process	6.25	1.18	8.49E-04	2.90E-02	2.74E-02	6
GO:0046394	carboxylic acid biosynthetic process	6.25	1.18	8.49E-04	2.90E-02	2.74E-02	6
GO:0022404	molting cycle process	5.21	0.83	1.10E-03	3.18E-02	3.01E-02	5
GO:0006032	chitin catabolic process	4.17	0.29	1.18E-04	8.05E-03	7.63E-03	4
GO:0009072	aromatic amino acid family metabolic process	4.17	0.29	1.18E-04	8.05E-03	7.63E-03	4
GO:0046348	amino sugar catabolic process	4.17	0.33	2.08E-04	1.12E-02	1.06E-02	4
GO:1901072	glucosamine-containing compound catabolic process	4.17	0.33	2.08E-04	1.12E-02	1.06E-02	4
GO:0006026	aminoglycan catabolic process	4.17	0.35	2.68E-04	1.26E-02	1.19E-02	4
GO:0006633	fatty acid biosynthetic process	4.17	0.41	5.22E-04	2.18E-02	2.06E-02	4
GO:0072330	monocarboxylic acid biosynthetic process	4.17	0.47	9.12E-04	2.98E-02	2.82E-02	4

GO:0030497	fatty acid elongation	3.13	0.20	7.86E-04	2.90E-02	2.74E-02	3
GO:0000038	very long-chain fatty acid metabolic process	3.13	0.22	1.07E-03	3.18E-02	3.01E-02	3
GO:0018990	ecdysis, chitin-based cuticle	3.13	0.24	1.40E-03	3.82E-02	3.62E-02	3
