# Supplementary material for: Transcriptomic changes during caste development through social interactions in the termite Zootermopsis nevadensis
Source: Ecol Evol. 2019 Feb 23;9(6):3446–56. doi: 10.1002/ece3.4976 (PMC6434549; doi:10.1002/ece3.4976)
Supplement: Supplementary file 18 [file ECE3-9-3446-s018.pdf]

Table S16. The enriched GO terms at Day 3 compared with Day 1–2 in the No. 1 larvae.

ID	Description	% in caste-DEG	% in all	pvalue	p.adjust	qvalue	Count
GO:0002009	morphogenesis of an epithelium	23.53	9.12	1.72E-06	9.99E-04	8.87E-04	28
GO:0048729	tissue morphogenesis	23.53	9.28	2.45E-06	9.99E-04	8.87E-04	28
GO:0009791	post-embryonic development	23.53	9.57	4.45E-06	1.36E-03	1.21E-03	28
GO:0002165	instar larval or pupal development	21.85	8.73	7.78E-06	1.90E-03	1.69E-03	26
GO:0035239	tube morphogenesis	19.33	7.51	1.84E-05	2.46E-03	2.18E-03	23
GO:0048569	post-embryonic animal organ development	18.49	6.76	1.10E-05	1.92E-03	1.70E-03	22
GO:0060562	epithelial tube morphogenesis	18.49	7.06	2.21E-05	2.46E-03	2.18E-03	22
GO:0007552	metamorphosis	18.49	7.59	6.74E-05	6.35E-03	5.63E-03	22
GO:0007444	imaginal disc development	18.49	8.57	3.94E-04	1.96E-02	1.74E-02	22
GO:0007560	imaginal disc morphogenesis	16.81	6.01	2.10E-05	2.46E-03	2.18E-03	20
GO:0048563	post-embryonic animal organ morphogenesis	16.81	6.01	2.10E-05	2.46E-03	2.18E-03	20
GO:0048707	instar larval or pupal morphogenesis	16.81	7.39	3.79E-04	1.96E-02	1.74E-02	20
GO:0009886	post-embryonic animal morphogenesis	16.81	7.57	5.22E-04	2.31E-02	2.05E-02	20
GO:0005975	carbohydrate metabolic process	14.29	5.50	2.32E-04	1.67E-02	1.48E-02	17
GO:0003002	regionalization	14.29	6.29	1.11E-03	3.66E-02	3.25E-02	17
GO:0035114	imaginal disc-derived appendage morphogenesis	13.45	5.11	3.13E-04	1.92E-02	1.70E-02	16
GO:0035120	post-embryonic appendage morphogenesis	13.45	5.11	3.13E-04	1.92E-02	1.70E-02	16
GO:0035107	appendage morphogenesis	13.45	5.17	3.59E-04	1.96E-02	1.74E-02	16
GO:0048737	imaginal disc-derived appendage development	13.45	5.25	4.27E-04	2.01E-02	1.79E-02	16
GO:0048736	appendage development	13.45	5.35	5.29E-04	2.31E-02	2.05E-02	16
GO:0007476	imaginal disc-derived wing morphogenesis	11.76	4.38	6.17E-04	2.43E-02	2.16E-02	14
GO:0007472	wing disc morphogenesis	11.76	4.48	7.76E-04	2.97E-02	2.63E-02	14
GO:0060541	respiratory system development	10.92	3.99	8.19E-04	3.04E-02	2.70E-02	13
GO:0042335	cuticle development	10.08	2.22	1.01E-05	1.92E-03	1.70E-03	12
GO:0007155	cell adhesion	9.24	2.87	5.47E-04	2.31E-02	2.05E-02	11

GO:0022610	biological adhesion	9.24	2.89	5.81E-04	2.37E-02	2.10E-02	11
GO:0007591	molting cycle, chitin-based cuticle	6.72	1.32	1.49E-04	1.22E-02	1.08E-02	8
GO:0042303	molting cycle	6.72	1.32	1.49E-04	1.22E-02	1.08E-02	8
GO:0035017	cuticle pattern formation	5.88	0.47	7.30E-07	8.93E-04	7.93E-04	7
GO:0009913	epidermal cell differentiation	5.88	0.88	6.42E-05	6.35E-03	5.63E-03	7
GO:0040003	chitin-based cuticle development	5.88	1.06	2.22E-04	1.67E-02	1.48E-02	7
GO:0008544	epidermis development	5.88	1.10	2.83E-04	1.92E-02	1.70E-02	7
GO:0035152	regulation of tube architecture, open tracheal system	5.88	1.45	1.51E-03	4.75E-02	4.21E-02	7
GO:0035315	hair cell differentiation	5.04	0.83	4.00E-04	1.96E-02	1.74E-02	6
GO:0035316	non-sensory hair organization	5.04	0.83	4.00E-04	1.96E-02	1.74E-02	6
GO:0035150	regulation of tube size	5.04	0.98	9.52E-04	3.33E-02	2.95E-02	6
GO:0009755	hormone-mediated signaling pathway	4.20	0.65	9.15E-04	3.29E-02	2.92E-02	5
GO:0071396	cellular response to lipid	4.20	0.67	1.06E-03	3.60E-02	3.19E-02	5
GO:0098742	cell-cell adhesion via plasma-membrane adhesion molecules	4.20	0.73	1.59E-03	4.85E-02	4.31E-02	5
GO:0045455	ecdysteroid metabolic process	3.36	0.43	1.42E-03	4.59E-02	4.07E-02	4
