# Supplementary material for: Transcriptomic changes during caste development through social interactions in the termite Zootermopsis nevadensis
Source: Ecol Evol. 2019 Feb 23;9(6):3446–56. doi: 10.1002/ece3.4976 (PMC6434549; doi:10.1002/ece3.4976)
Supplement: Supplementary file 19 [file ECE3-9-3446-s019.pdf]

Table S17. The enriched GO terms in the No. 1 larva compared with the No. 2 larva at Day 1–2.

ID	Description	% in caste-DEG	% in all	pvalue	p.adjust	qvalue	Count
GO:0022402	cell cycle process	32.73	7.78	2.03E-21	6.89E-19	6.07E-19	54
GO:0000278	mitotic cell cycle	30.91	7.45	7.29E-20	1.98E-17	1.75E-17	51
GO:0051276	chromosome organization	29.09	7.17	3.11E-18	6.05E-16	5.33E-16	48
GO:0006259	DNA metabolic process	24.24	3.93	6.19E-22	2.81E-19	2.47E-19	40
GO:1903047	mitotic cell cycle process	24.24	5.29	5.23E-17	5.93E-15	5.23E-15	40
GO:0000280	nuclear division	21.21	3.77	7.81E-18	1.33E-15	1.17E-15	35
GO:0048285	organelle fission	21.21	3.95	3.83E-17	4.74E-15	4.17E-15	35
GO:0006260	DNA replication	20.00	1.49	1.81E-30	2.46E-27	2.17E-27	33
GO:0051726	regulation of cell cycle	20.00	5.29	1.20E-11	8.59E-10	7.57E-10	33
GO:0006261	DNA-dependent DNA replication	18.18	1.24	4.04E-29	2.75E-26	2.42E-26	30
GO:0007292	female gamete generation	17.58	10.04	1.72E-03	2.04E-02	1.80E-02	29
GO:0007010	cytoskeleton organization	16.97	8.35	1.92E-04	3.18E-03	2.80E-03	28
GO:0007017	microtubule-based process	15.15	5.46	2.29E-06	6.63E-05	5.84E-05	25
GO:0010564	regulation of cell cycle process	14.55	3.03	7.33E-11	4.34E-09	3.82E-09	24
GO:0045165	cell fate commitment	14.55	5.99	3.65E-05	7.10E-04	6.25E-04	24
GO:0140014	mitotic nuclear division	13.94	2.04	7.47E-14	7.26E-12	6.40E-12	23
GO:0051321	meiotic cell cycle	13.94	2.42	3.80E-12	3.23E-10	2.85E-10	23
GO:0006974	cellular response to DNA damage stimulus	13.94	4.54	1.07E-06	3.39E-05	2.98E-05	23
GO:0006323	DNA packaging	13.33	1.32	3.27E-17	4.45E-15	3.92E-15	22
GO:0071103	DNA conformation change	13.33	1.63	4.75E-15	4.97E-13	4.38E-13	22
GO:1903046	meiotic cell cycle process	13.33	2.26	6.85E-12	5.18E-10	4.56E-10	22
GO:0007059	chromosome segregation	13.33	2.34	1.45E-11	9.84E-10	8.67E-10	22
GO:0051301	cell division	13.33	3.71	1.16E-07	4.89E-06	4.31E-06	22
GO:0098813	nuclear chromosome segregation	12.73	2.02	5.36E-12	4.29E-10	3.78E-10	21
GO:0007346	regulation of mitotic cell cycle	12.73	3.44	1.44E-07	5.28E-06	4.65E-06	21

GO:0000226	microtubule cytoskeleton organization	12.73	4.21	4.30E-06	1.15E-04	1.01E-04	21
GO:0140013	meiotic nuclear division	12.12	2.12	1.19E-10	6.76E-09	5.96E-09	20
GO:0006325	chromatin organization	11.52	4.68	2.17E-04	3.40E-03	3.00E-03	19
GO:0030261	chromosome condensation	10.91	0.77	2.20E-17	3.33E-15	2.93E-15	18
GO:0000819	sister chromatid segregation	10.91	1.53	2.15E-11	1.39E-09	1.22E-09	18
GO:0006281	DNA repair	10.91	2.30	2.51E-08	1.31E-06	1.16E-06	18
GO:0045786	negative regulation of cell cycle	10.91	2.36	3.83E-08	1.80E-06	1.58E-06	18
GO:0000070	mitotic sister chromatid segregation	10.30	1.36	3.05E-11	1.89E-09	1.66E-09	17
GO:0006270	DNA replication initiation	9.09	0.43	1.89E-18	4.29E-16	3.78E-16	15
GO:0000075	cell cycle checkpoint	9.09	1.83	2.12E-07	7.61E-06	6.70E-06	15
GO:0044772	mitotic cell cycle phase transition	8.48	2.57	7.00E-05	1.25E-03	1.10E-03	14
GO:0044770	cell cycle phase transition	8.48	2.59	7.64E-05	1.35E-03	1.19E-03	14
GO:0007051	spindle organization	7.88	1.61	1.66E-06	5.02E-05	4.42E-05	13
GO:1901987	regulation of cell cycle phase transition	7.88	2.34	1.07E-04	1.87E-03	1.65E-03	13
GO:0044786	cell cycle DNA replication	7.27	0.47	1.36E-12	1.24E-10	1.09E-10	12
GO:0006310	DNA recombination	7.27	0.98	3.38E-08	1.64E-06	1.45E-06	12
GO:0045930	negative regulation of mitotic cell cycle	7.27	1.87	4.86E-05	9.32E-04	8.21E-04	12
GO:0010948	negative regulation of cell cycle process	7.27	1.89	5.42E-05	1.02E-03	9.02E-04	12
GO:1901990	regulation of mitotic cell cycle phase transition	7.27	2.32	3.89E-04	5.70E-03	5.02E-03	12
GO:0007052	mitotic spindle organization	6.67	0.90	1.29E-07	4.89E-06	4.31E-06	11
GO:0051783	regulation of nuclear division	6.67	0.90	1.29E-07	4.89E-06	4.31E-06	11
GO:1902850	microtubule cytoskeleton organization involved in mitosis	6.67	1.22	3.62E-06	1.03E-04	9.05E-05	11
GO:0000910	cytokinesis	6.67	1.61	5.49E-05	1.02E-03	9.02E-04	11
GO:0071897	DNA biosynthetic process	6.06	0.63	3.36E-08	1.64E-06	1.45E-06	10
GO:0051052	regulation of DNA metabolic process	6.06	0.71	1.24E-07	4.89E-06	4.31E-06	10
GO:0007127	meiosis I	6.06	0.83	6.35E-07	2.11E-05	1.86E-05	10
GO:0006302	double-strand break repair	6.06	0.92	1.61E-06	4.98E-05	4.38E-05	10

GO:0007098	centrosome cycle	6.06	1.08	7.79E-06	1.93E-04	1.70E-04	10
GO:0031023	microtubule organizing center organization	6.06	1.14	1.30E-05	3.06E-04	2.70E-04	10
GO:0061640	cytoskeleton-dependent cytokinesis	6.06	1.55	1.96E-04	3.21E-03	2.83E-03	10
GO:0007093	mitotic cell cycle checkpoint	6.06	1.59	2.44E-04	3.68E-03	3.24E-03	10
GO:0001709	cell fate determination	6.06	2.06	1.91E-03	2.23E-02	1.96E-02	10
GO:0007088	regulation of mitotic nuclear division	5.45	0.83	6.09E-06	1.56E-04	1.38E-04	9
GO:0045787	positive regulation of cell cycle	5.45	0.94	1.66E-05	3.42E-04	3.01E-04	9
GO:0008356	asymmetric cell division	5.45	1.63	1.31E-03	1.63E-02	1.44E-02	9
GO:1901988	negative regulation of cell cycle phase transition	5.45	1.69	1.70E-03	2.03E-02	1.79E-02	9
GO:1901991	negative regulation of mitotic cell cycle phase transition	5.45	1.69	1.70E-03	2.03E-02	1.79E-02	9
GO:0007076	mitotic chromosome condensation	4.85	0.31	7.22E-09	3.93E-07	3.46E-07	8
GO:0000724	double-strand break repair via homologous recombination	4.85	0.61	4.26E-06	1.15E-04	1.01E-04	8
GO:0000725	recombinational repair	4.85	0.61	4.26E-06	1.15E-04	1.01E-04	8
GO:0051304	chromosome separation	4.85	0.65	7.23E-06	1.82E-04	1.61E-04	8
GO:0045132	meiotic chromosome segregation	4.85	0.71	1.48E-05	3.31E-04	2.91E-04	8
GO:0007143	female meiotic nuclear division	4.85	0.75	2.30E-05	4.67E-04	4.11E-04	8
GO:0017145	stem cell division	4.85	1.45	2.40E-03	2.68E-02	2.36E-02	8
GO:0031570	DNA integrity checkpoint	4.85	1.53	3.40E-03	3.61E-02	3.18E-02	8
GO:0006271	DNA strand elongation involved in DNA replication	4.24	0.26	6.16E-08	2.70E-06	2.38E-06	7
GO:0022616	DNA strand elongation	4.24	0.26	6.16E-08	2.70E-06	2.38E-06	7
GO:0051784	negative regulation of nuclear division	4.24	0.29	1.20E-07	4.89E-06	4.31E-06	7
GO:0042023	DNA endoreduplication	4.24	0.31	2.18E-07	7.61E-06	6.71E-06	7
GO:0006277	DNA amplification	4.24	0.35	6.23E-07	2.11E-05	1.86E-05	7
GO:0051302	regulation of cell division	4.24	0.41	2.28E-06	6.63E-05	5.84E-05	7
GO:0045448	mitotic cell cycle, embryonic	4.24	0.51	1.23E-05	2.93E-04	2.58E-04	7
GO:0000281	mitotic cytokinesis	4.24	0.98	9.79E-04	1.23E-02	1.09E-02	7
GO:0007140	male meiotic nuclear division	4.24	0.98	9.79E-04	1.23E-02	1.09E-02	7

GO:0007306	eggshell chorion assembly	4.24	1.12	2.21E-03	2.49E-02	2.19E-02	7
GO:0010639	negative regulation of organelle organization	4.24	1.12	2.21E-03	2.49E-02	2.19E-02	7
GO:0007419	ventral cord development	4.24	1.14	2.46E-03	2.72E-02	2.40E-02	7
GO:0048565	digestive tract development	4.24	1.26	4.41E-03	4.51E-02	3.98E-02	7
GO:0055123	digestive system development	4.24	1.26	4.41E-03	4.51E-02	3.98E-02	7
GO:0006275	regulation of DNA replication	3.64	0.24	1.03E-06	3.32E-05	2.93E-05	6
GO:0007307	eggshell chorion gene amplification	3.64	0.31	5.11E-06	1.34E-04	1.18E-04	6
GO:0051299	centrosome separation	3.64	0.41	3.44E-05	6.78E-04	5.97E-04	6
GO:0090068	positive regulation of cell cycle process	3.64	0.57	2.67E-04	4.00E-03	3.52E-03	6
GO:0045931	positive regulation of mitotic cell cycle	3.64	0.63	4.80E-04	6.81E-03	6.00E-03	6
GO:0006333	chromatin assembly or disassembly	3.64	0.65	5.75E-04	7.67E-03	6.76E-03	6
GO:0065004	protein-DNA complex assembly	3.64	0.71	9.47E-04	1.22E-02	1.07E-02	6
GO:0018105	peptidyl-serine phosphorylation	3.64	0.79	1.70E-03	2.03E-02	1.79E-02	6
GO:0018209	peptidyl-serine modification	3.64	0.81	1.95E-03	2.25E-02	1.98E-02	6
GO:0032465	regulation of cytokinesis	3.03	0.20	8.85E-06	2.15E-04	1.90E-04	5
GO:0031577	spindle checkpoint	3.03	0.22	1.58E-05	3.31E-04	2.91E-04	5
GO:0033048	negative regulation of mitotic sister chromatid segregation	3.03	0.22	1.58E-05	3.31E-04	2.91E-04	5
GO:0045841	negative regulation of mitotic metaphase/anaphase transition	3.03	0.22	1.58E-05	3.31E-04	2.91E-04	5
GO:1902100	negative regulation of metaphase/anaphase transition of cell cycle	3.03	0.22	1.58E-05	3.31E-04	2.91E-04	5
GO:1905819	negative regulation of chromosome separation	3.03	0.22	1.58E-05	3.31E-04	2.91E-04	5
GO:2000816	negative regulation of mitotic sister chromatid separation	3.03	0.22	1.58E-05	3.31E-04	2.91E-04	5
GO:0045839	negative regulation of mitotic nuclear division	3.03	0.24	2.63E-05	5.27E-04	4.64E-04	5
GO:0033046	negative regulation of sister chromatid segregation	3.03	0.29	6.30E-05	1.14E-03	1.01E-03	5
GO:0051985	negative regulation of chromosome segregation	3.03	0.29	6.30E-05	1.14E-03	1.01E-03	5
GO:0007131	reciprocal meiotic recombination	3.03	0.33	1.30E-04	2.24E-03	1.98E-03	5
GO:0033301	cell cycle comprising mitosis without cytokinesis	3.03	0.35	1.79E-04	3.02E-03	2.66E-03	5
GO:0035825	homologous recombination	3.03	0.35	1.79E-04	3.02E-03	2.66E-03	5

GO:0007494	midgut development	3.03	0.37	2.42E-04	3.68E-03	3.24E-03	5
GO:0030071	regulation of mitotic metaphase/anaphase transition	3.03	0.41	4.14E-04	5.93E-03	5.23E-03	5
GO:1902099	regulation of metaphase/anaphase transition of cell cycle	3.03	0.41	4.14E-04	5.93E-03	5.23E-03	5
GO:0007091	metaphase/anaphase transition of mitotic cell cycle	3.03	0.43	5.29E-04	7.13E-03	6.28E-03	5
GO:0010965	regulation of mitotic sister chromatid separation	3.03	0.43	5.29E-04	7.13E-03	6.28E-03	5
GO:0044784	metaphase/anaphase transition of cell cycle	3.03	0.43	5.29E-04	7.13E-03	6.28E-03	5
GO:1905818	regulation of chromosome separation	3.03	0.43	5.29E-04	7.13E-03	6.28E-03	5
GO:0033047	regulation of mitotic sister chromatid segregation	3.03	0.45	6.66E-04	8.72E-03	7.68E-03	5
GO:0051306	mitotic sister chromatid separation	3.03	0.45	6.66E-04	8.72E-03	7.68E-03	5
GO:0033045	regulation of sister chromatid segregation	3.03	0.53	1.49E-03	1.85E-02	1.63E-02	5
GO:0051983	regulation of chromosome segregation	3.03	0.55	1.78E-03	2.09E-02	1.84E-02	5
GO:2001251	negative regulation of chromosome organization	3.03	0.59	2.48E-03	2.73E-02	2.40E-02	5
GO:0031109	microtubule polymerization or depolymerization	3.03	0.61	2.90E-03	3.13E-02	2.76E-02	5
GO:0042078	germ-line stem cell division	3.03	0.65	3.88E-03	4.04E-02	3.55E-02	5
GO:0007094	mitotic spindle assembly checkpoint	2.42	0.20	2.20E-04	3.40E-03	3.00E-03	4
GO:0007100	mitotic centrosome separation	2.42	0.20	2.20E-04	3.40E-03	3.00E-03	4
GO:0071173	spindle assembly checkpoint	2.42	0.20	2.20E-04	3.40E-03	3.00E-03	4
GO:0071174	mitotic spindle checkpoint	2.42	0.20	2.20E-04	3.40E-03	3.00E-03	4
GO:0000018	regulation of DNA recombination	2.42	0.22	3.37E-04	4.98E-03	4.39E-03	4
GO:0050000	chromosome localization	2.42	0.24	4.92E-04	6.90E-03	6.08E-03	4
GO:0035186	syncytial blastoderm mitotic cell cycle	2.42	0.29	9.44E-04	1.22E-02	1.07E-02	4
GO:0006334	nucleosome assembly	2.42	0.33	1.63E-03	2.00E-02	1.76E-02	4
GO:0044719	regulation of imaginal disc-derived wing size	2.42	0.35	2.07E-03	2.37E-02	2.09E-02	4
GO:0098728	germline stem cell asymmetric division	2.42	0.37	2.60E-03	2.83E-02	2.49E-02	4
GO:1901989	positive regulation of cell cycle phase transition	2.42	0.39	3.21E-03	3.44E-02	3.03E-02	4
GO:0016321	female meiosis chromosome segregation	2.42	0.43	4.70E-03	4.74E-02	4.17E-02	4
GO:0072528	pyrimidine-containing compound biosynthetic process	2.42	0.43	4.70E-03	4.74E-02	4.17E-02	4

GO:0006298	mismatch repair	1.82	0.20	3.75E-03	3.93E-02	3.46E-02	3
GO:0035223	leg disc pattern formation	1.82	0.20	3.75E-03	3.93E-02	3.46E-02	3
