# Supplementary material for: Transcriptomic changes during caste development through social interactions in the termite Zootermopsis nevadensis
Source: Ecol Evol. 2019 Feb 23;9(6):3446–56. doi: 10.1002/ece3.4976 (PMC6434549; doi:10.1002/ece3.4976)
Supplement: Supplementary file 20 [file ECE3-9-3446-s020.pdf]

Table S18. The enriched GO terms in the No. 2 larva compared with the No. 1 larva at Day 1-2.

ID	Description	% in caste-DEG	% in all	pvalue	p.adjust	qvalue	Count
GO:0006040	amino sugar metabolic process	12.00	0.77	4.91E-15	2.49E-12	2.43E-12	15
GO:0042335	cuticle development	12.00	2.22	7.05E-08	8.92E-06	8.72E-06	15
GO:0006030	chitin metabolic process	11.20	0.59	1.29E-15	1.30E-12	1.27E-12	14
GO:1901071	glucosamine-containing compound metabolic process	11.20	0.71	3.38E-14	1.14E-11	1.12E-11	14
GO:0006022	aminoglycan metabolic process	11.20	0.96	3.85E-12	9.76E-10	9.54E-10	14
GO:0048066	developmental pigmentation	8.00	1.65	3.23E-05	1.72E-03	1.68E-03	10
GO:0043473	pigmentation	8.00	1.69	4.01E-05	2.03E-03	1.98E-03	10
GO:0040003	chitin-based cuticle development	7.20	1.06	4.90E-06	4.14E-04	4.05E-04	9
GO:0007591	molting cycle, chitin-based cuticle	7.20	1.32	3.22E-05	1.72E-03	1.68E-03	9
GO:0042303	molting cycle	7.20	1.32	3.22E-05	1.72E-03	1.68E-03	9
GO:1901615	organic hydroxy compound metabolic process	7.20	1.85	4.53E-04	1.64E-02	1.60E-02	9
GO:0048067	cuticle pigmentation	6.40	0.73	2.34E-06	2.15E-04	2.11E-04	8
GO:0022404	molting cycle process	6.40	0.83	6.64E-06	5.17E-04	5.06E-04	8
GO:0006032	chitin catabolic process	5.60	0.29	1.73E-08	3.51E-06	3.43E-06	7
GO:0046348	amino sugar catabolic process	5.60	0.33	5.53E-08	8.00E-06	7.82E-06	7
GO:1901072	glucosamine-containing compound catabolic process	5.60	0.33	5.53E-08	8.00E-06	7.82E-06	7
GO:0006026	aminoglycan catabolic process	5.60	0.35	9.20E-08	1.04E-05	1.01E-05	7
GO:1901136	carbohydrate derivative catabolic process	5.60	0.67	1.44E-05	1.04E-03	1.02E-03	7
GO:1901617	organic hydroxy compound biosynthetic process	5.60	0.69	1.77E-05	1.20E-03	1.17E-03	7
GO:0035151	regulation of tube size, open tracheal system	5.60	0.94	1.37E-04	6.31E-03	6.17E-03	7
GO:0018958	phenol-containing compound metabolic process	5.60	0.98	1.81E-04	7.32E-03	7.16E-03	7
GO:0035150	regulation of tube size	5.60	0.98	1.81E-04	7.32E-03	7.16E-03	7
GO:0046148	pigment biosynthetic process	5.60	1.04	2.67E-04	1.04E-02	1.02E-02	7
GO:0019748	secondary metabolic process	5.60	1.16	5.36E-04	1.87E-02	1.83E-02	7
GO:0046189	phenol-containing compound biosynthetic process	4.80	0.26	3.57E-07	3.62E-05	3.54E-05	6

GO:0044550	secondary metabolite biosynthetic process	4.80	0.49	2.23E-05	1.41E-03	1.38E-03	6
GO:0006582	melanin metabolic process	4.80	0.77	3.41E-04	1.28E-02	1.25E-02	6
GO:0008362	chitin-based embryonic cuticle biosynthetic process	4.00	0.39	8.60E-05	4.15E-03	4.06E-03	5
GO:0018990	ecdysis, chitin-based cuticle	3.20	0.24	1.69E-04	7.32E-03	7.16E-03	4
