# Supplementary material for: Transcriptomic changes during caste development through social interactions in the termite Zootermopsis nevadensis
Source: Ecol Evol. 2019 Feb 23;9(6):3446–56. doi: 10.1002/ece3.4976 (PMC6434549; doi:10.1002/ece3.4976)
Supplement: Supplementary file 21 [file ECE3-9-3446-s021.pdf]

Table S19. The enriched GO terms in the No. 1 larva compared with the No. 2 larva at Day 3.

ID	Description	% in caste-DEG	% in all	pvalue	p.adjust	qvalue	Count
GO:0000278	mitotic cell cycle	19.56	7.45	2.70E-13	1.61E-10	1.31E-10	62
GO:0022402	cell cycle process	18.93	7.78	2.05E-11	8.63E-09	7.01E-09	60
GO:0002009	morphogenesis of an epithelium	17.98	9.12	2.21E-07	1.80E-05	1.46E-05	57
GO:0048729	tissue morphogenesis	17.98	9.28	4.07E-07	3.17E-05	2.57E-05	57
GO:0009791	post-embryonic development	17.67	9.57	2.54E-06	1.49E-04	1.21E-04	56
GO:0051276	chromosome organization	17.03	7.17	6.24E-10	8.59E-08	6.98E-08	54
GO:0002165	instar larval or pupal development	16.72	8.73	1.63E-06	1.08E-04	8.76E-05	53
GO:0007292	female gamete generation	16.40	10.04	1.81E-04	3.77E-03	3.06E-03	52
GO:0007444	imaginal disc development	15.46	8.57	2.29E-05	7.72E-04	6.27E-04	49
GO:0035239	tube morphogenesis	14.83	7.51	2.95E-06	1.64E-04	1.33E-04	47
GO:0007552	metamorphosis	14.83	7.59	4.01E-06	2.05E-04	1.66E-04	47
GO:0048477	oogenesis	14.51	9.36	1.43E-03	1.71E-02	1.39E-02	46
GO:1903047	mitotic cell cycle process	14.20	5.29	3.93E-10	6.45E-08	5.24E-08	45
GO:0060562	epithelial tube morphogenesis	14.20	7.06	3.02E-06	1.64E-04	1.33E-04	45
GO:0000904	cell morphogenesis involved in differentiation	14.20	7.90	5.63E-05	1.60E-03	1.30E-03	45
GO:0051726	regulation of cell cycle	13.88	5.29	1.33E-09	1.70E-07	1.38E-07	44
GO:0007423	sensory organ development	13.88	7.15	9.72E-06	3.93E-04	3.19E-04	44
GO:0048707	instar larval or pupal morphogenesis	13.56	7.39	5.07E-05	1.46E-03	1.19E-03	43
GO:0009886	post-embryonic animal morphogenesis	13.56	7.57	9.09E-05	2.11E-03	1.72E-03	43
GO:0007010	cytoskeleton organization	13.56	8.35	7.89E-04	1.10E-02	8.96E-03	43
GO:0031175	neuron projection development	13.56	9.00	3.50E-03	3.25E-02	2.64E-02	43
GO:0048646	anatomical structure formation involved in morphogenesis	13.25	7.31	8.37E-05	2.07E-03	1.68E-03	42
GO:0009790	embryo development	13.25	7.49	1.48E-04	3.31E-03	2.69E-03	42
GO:2000026	regulation of multicellular organismal development	13.25	7.63	2.25E-04	4.43E-03	3.60E-03	42
GO:0048569	post-embryonic animal organ development	12.93	6.76	2.91E-05	8.81E-04	7.16E-04	41

GO:0006259	DNA metabolic process	12.30	3.93	5.62E-11	1.26E-08	1.02E-08	39
GO:0007560	imaginal disc morphogenesis	12.30	6.01	9.34E-06	3.93E-04	3.19E-04	39
GO:0048563	post-embryonic animal organ morphogenesis	12.30	6.01	9.34E-06	3.93E-04	3.19E-04	39
GO:0010605	negative regulation of macromolecule metabolic process	12.30	8.00	3.84E-03	3.53E-02	2.86E-02	39
GO:0048667	cell morphogenesis involved in neuron differentiation	11.99	7.17	9.41E-04	1.27E-02	1.03E-02	38
GO:0048736	appendage development	11.36	5.35	9.66E-06	3.93E-04	3.19E-04	36
GO:0007389	pattern specification process	11.36	6.86	1.56E-03	1.84E-02	1.49E-02	36
GO:0000280	nuclear division	11.04	3.77	3.82E-09	4.27E-07	3.47E-07	35
GO:0048285	organelle fission	11.04	3.95	1.40E-08	1.40E-06	1.13E-06	35
GO:0048737	imaginal disc-derived appendage development	11.04	5.25	1.58E-05	6.03E-04	4.90E-04	35
GO:0001654	eye development	11.04	5.78	1.22E-04	2.80E-03	2.27E-03	35
GO:0030855	epithelial cell differentiation	11.04	5.88	1.73E-04	3.73E-03	3.03E-03	35
GO:0045165	cell fate commitment	11.04	5.99	2.44E-04	4.69E-03	3.81E-03	35
GO:0035114	imaginal disc-derived appendage morphogenesis	10.73	5.11	2.18E-05	7.51E-04	6.10E-04	34
GO:0035120	post-embryonic appendage morphogenesis	10.73	5.11	2.18E-05	7.51E-04	6.10E-04	34
GO:0035107	appendage morphogenesis	10.73	5.17	2.81E-05	8.68E-04	7.05E-04	34
GO:0048749	compound eye development	10.73	5.46	8.58E-05	2.07E-03	1.68E-03	34
GO:0003002	regionalization	10.73	6.29	1.27E-03	1.60E-02	1.30E-02	34
GO:0035220	wing disc development	10.41	6.09	1.42E-03	1.70E-02	1.38E-02	33
GO:0051172	negative regulation of nitrogen compound metabolic process	10.41	6.37	3.02E-03	2.93E-02	2.38E-02	33
GO:0010629	negative regulation of gene expression	10.41	6.43	3.52E-03	3.25E-02	2.64E-02	33
GO:0005975	carbohydrate metabolic process	9.78	5.50	1.04E-03	1.36E-02	1.11E-02	31
GO:0045595	regulation of cell differentiation	9.46	5.19	8.37E-04	1.14E-02	9.29E-03	30
GO:0007017	microtubule-based process	9.46	5.46	1.87E-03	2.07E-02	1.68E-02	30
GO:0010564	regulation of cell cycle process	9.15	3.03	4.72E-08	4.22E-06	3.43E-06	29
GO:0048592	eye morphogenesis	9.15	4.62	2.58E-04	4.80E-03	3.90E-03	29
GO:0090596	sensory organ morphogenesis	9.15	4.62	2.58E-04	4.80E-03	3.90E-03	29

GO:0006260	DNA replication	8.83	1.49	1.29E-15	2.31E-12	1.87E-12	28
GO:0007059	chromosome segregation	8.83	2.34	3.97E-10	6.45E-08	5.24E-08	28
GO:0007346	regulation of mitotic cell cycle	8.83	3.44	2.58E-06	1.49E-04	1.21E-04	28
GO:0045934	negative regulation of nucleobase-containing compound metabolic process	8.83	4.11	7.97E-05	2.05E-03	1.67E-03	28
GO:0007476	imaginal disc-derived wing morphogenesis	8.83	4.38	2.38E-04	4.63E-03	3.76E-03	28
GO:0001745	compound eye morphogenesis	8.83	4.44	3.01E-04	5.50E-03	4.47E-03	28
GO:0007472	wing disc morphogenesis	8.83	4.48	3.51E-04	5.95E-03	4.84E-03	28
GO:0010558	negative regulation of macromolecule biosynthetic process	8.52	4.62	1.29E-03	1.60E-02	1.30E-02	27
GO:2000113	negative regulation of cellular macromolecule biosynthetic process	8.52	4.62	1.29E-03	1.60E-02	1.30E-02	27
GO:0006325	chromatin organization	8.52	4.68	1.57E-03	1.84E-02	1.49E-02	27
GO:0009890	negative regulation of biosynthetic process	8.52	4.80	2.30E-03	2.39E-02	1.94E-02	27
GO:0031327	negative regulation of cellular biosynthetic process	8.52	4.80	2.30E-03	2.39E-02	1.94E-02	27
GO:0008283	cell proliferation	8.52	4.91	3.10E-03	2.97E-02	2.41E-02	27
GO:0098813	nuclear chromosome segregation	8.20	2.02	2.89E-10	5.74E-08	4.66E-08	26
GO:0007417	central nervous system development	8.20	3.44	2.36E-05	7.81E-04	6.34E-04	26
GO:0045892	negative regulation of transcription, DNA-templated	8.20	3.71	8.67E-05	2.07E-03	1.68E-03	26
GO:1902679	negative regulation of RNA biosynthetic process	8.20	3.71	8.67E-05	2.07E-03	1.68E-03	26
GO:1903507	negative regulation of nucleic acid-templated transcription	8.20	3.71	8.67E-05	2.07E-03	1.68E-03	26
GO:0051253	negative regulation of RNA metabolic process	8.20	3.85	1.64E-04	3.62E-03	2.94E-03	26
GO:0006261	DNA-dependent DNA replication	7.89	1.24	7.36E-15	6.59E-12	5.35E-12	25
GO:0140014	mitotic nuclear division	7.89	2.04	2.00E-09	2.38E-07	1.93E-07	25
GO:0061061	muscle structure development	7.89	3.97	6.54E-04	9.51E-03	7.72E-03	25
GO:0000226	microtubule cytoskeleton organization	7.89	4.21	1.57E-03	1.84E-02	1.49E-02	25
GO:0045935	positive regulation of nucleobase-containing compound metabolic process	7.89	4.62	5.48E-03	4.58E-02	3.72E-02	25
GO:0045786	negative regulation of cell cycle	7.57	2.36	2.18E-07	1.80E-05	1.46E-05	24
GO:0022603	regulation of anatomical structure morphogenesis	7.57	3.64	4.33E-04	6.85E-03	5.57E-03	24
GO:0071103	DNA conformation change	7.26	1.63	4.51E-10	6.73E-08	5.47E-08	23

GO:0007163	establishment or maintenance of cell polarity	7.26	3.36	3.22E-04	5.83E-03	4.73E-03	23
GO:0016358	dendrite development	7.26	3.38	3.53E-04	5.95E-03	4.84E-03	23
GO:0006323	DNA packaging	6.94	1.32	3.05E-11	8.63E-09	7.01E-09	22
GO:0051321	meiotic cell cycle	6.94	2.42	5.17E-06	2.50E-04	2.03E-04	22
GO:0048813	dendrite morphogenesis	6.94	3.20	4.03E-04	6.50E-03	5.28E-03	22
GO:0051301	cell division	6.94	3.71	2.93E-03	2.87E-02	2.33E-02	22
GO:0007411	axon guidance	6.94	3.91	5.64E-03	4.65E-02	3.78E-02	22
GO:0000819	sister chromatid segregation	6.62	1.53	4.53E-09	4.77E-07	3.87E-07	21
GO:1903046	meiotic cell cycle process	6.62	2.26	5.83E-06	2.75E-04	2.23E-04	21
GO:0000070	mitotic sister chromatid segregation	5.99	1.36	2.01E-08	1.89E-06	1.53E-06	19
GO:0001738	morphogenesis of a polarized epithelium	5.99	1.85	3.60E-06	1.90E-04	1.54E-04	19
GO:0140013	meiotic nuclear division	5.99	2.12	2.75E-05	8.68E-04	7.05E-04	19
GO:0001700	embryonic development via the syncytial blastoderm	5.99	2.79	1.13E-03	1.46E-02	1.19E-02	19
GO:0009792	embryo development ending in birth or egg hatching	5.99	2.83	1.35E-03	1.63E-02	1.33E-02	19
GO:0007155	cell adhesion	5.99	2.87	1.60E-03	1.86E-02	1.51E-02	19
GO:0022610	biological adhesion	5.99	2.89	1.74E-03	1.95E-02	1.58E-02	19
GO:0048732	gland development	5.99	3.11	4.12E-03	3.64E-02	2.95E-02	19
GO:0001736	establishment of planar polarity	5.68	1.63	2.10E-06	1.29E-04	1.05E-04	18
GO:0007164	establishment of tissue polarity	5.68	1.63	2.10E-06	1.29E-04	1.05E-04	18
GO:0042335	cuticle development	5.68	2.22	1.75E-04	3.73E-03	3.03E-03	18
GO:0019318	hexose metabolic process	5.68	2.99	6.01E-03	4.91E-02	3.99E-02	18
GO:0030261	chromosome condensation	5.36	0.77	3.19E-11	8.63E-09	7.01E-09	17
GO:0010948	negative regulation of cell cycle process	5.36	1.89	7.25E-05	2.00E-03	1.62E-03	17
GO:0007431	salivary gland development	5.36	2.40	1.34E-03	1.63E-02	1.32E-02	17
GO:0035272	exocrine system development	5.36	2.40	1.34E-03	1.63E-02	1.32E-02	17
GO:0044772	mitotic cell cycle phase transition	5.36	2.57	2.75E-03	2.71E-02	2.20E-02	17
GO:0044770	cell cycle phase transition	5.36	2.59	2.99E-03	2.91E-02	2.37E-02	17

GO:0000075	cell cycle checkpoint	5.05	1.83	1.66E-04	3.63E-03	2.95E-03	16
GO:0007517	muscle organ development	5.05	2.10	8.07E-04	1.11E-02	9.02E-03	16
GO:0006281	DNA repair	5.05	2.30	2.21E-03	2.38E-02	1.93E-02	16
GO:1901987	regulation of cell cycle phase transition	5.05	2.34	2.65E-03	2.64E-02	2.14E-02	16
GO:0035218	leg disc development	4.73	1.57	9.09E-05	2.11E-03	1.72E-03	15
GO:0045930	negative regulation of mitotic cell cycle	4.73	1.87	6.96E-04	9.97E-03	8.10E-03	15
GO:0051129	negative regulation of cellular component organization	4.73	2.22	3.97E-03	3.59E-02	2.91E-02	15
GO:0022604	regulation of cell morphogenesis	4.73	2.26	4.73E-03	4.01E-02	3.26E-02	15
GO:1901990	regulation of mitotic cell cycle phase transition	4.73	2.32	6.08E-03	4.94E-02	4.01E-02	15
GO:0042067	establishment of ommatidial planar polarity	4.42	1.00	1.40E-06	9.63E-05	7.82E-05	14
GO:0008544	epidermis development	4.42	1.10	5.00E-06	2.49E-04	2.02E-04	14
GO:0048565	digestive tract development	4.42	1.26	2.79E-05	8.68E-04	7.05E-04	14
GO:0055123	digestive system development	4.42	1.26	2.79E-05	8.68E-04	7.05E-04	14
GO:0060322	head development	4.42	1.83	1.67E-03	1.89E-02	1.54E-02	14
GO:0016055	Wnt signaling pathway	4.42	1.97	3.43E-03	3.25E-02	2.64E-02	14
GO:1905114	cell surface receptor signaling pathway involved in cell-cell signaling	4.42	2.04	4.55E-03	3.88E-02	3.15E-02	14
GO:0006270	DNA replication initiation	4.10	0.43	3.38E-11	8.63E-09	7.01E-09	13
GO:0007480	imaginal disc-derived leg morphogenesis	3.79	1.30	6.61E-04	9.54E-03	7.75E-03	12
GO:0007478	leg disc morphogenesis	3.79	1.34	8.82E-04	1.20E-02	9.71E-03	12
GO:0035152	regulation of tube architecture, open tracheal system	3.79	1.45	1.71E-03	1.93E-02	1.57E-02	12
GO:0007420	brain development	3.79	1.57	3.47E-03	3.25E-02	2.64E-02	12
GO:0008038	neuron recognition	3.79	1.57	3.47E-03	3.25E-02	2.64E-02	12
GO:0000910	cytokinesis	3.79	1.61	4.31E-03	3.71E-02	3.01E-02	12
GO:0007051	spindle organization	3.79	1.61	4.31E-03	3.71E-02	3.01E-02	12
GO:0008037	cell recognition	3.79	1.61	4.31E-03	3.71E-02	3.01E-02	12
GO:0072089	stem cell proliferation	3.79	1.61	4.31E-03	3.71E-02	3.01E-02	12
GO:0045132	meiotic chromosome segregation	3.47	0.71	6.99E-06	3.21E-04	2.61E-04	11

GO:0009913	epidermal cell differentiation	3.47	0.88	6.04E-05	1.69E-03	1.37E-03	11
GO:0051783	regulation of nuclear division	3.47	0.90	7.59E-05	2.03E-03	1.65E-03	11
GO:0035150	regulation of tube size	3.47	0.98	1.77E-04	3.73E-03	3.03E-03	11
GO:0010639	negative regulation of organelle organization	3.47	1.12	6.25E-04	9.16E-03	7.44E-03	11
GO:0007419	ventral cord development	3.47	1.14	7.33E-04	1.04E-02	8.46E-03	11
GO:1902850	microtubule cytoskeleton organization involved in mitosis	3.47	1.22	1.34E-03	1.63E-02	1.32E-02	11
GO:0035017	cuticle pattern formation	3.15	0.47	5.86E-07	4.20E-05	3.41E-05	10
GO:0044786	cell cycle DNA replication	3.15	0.47	5.86E-07	4.20E-05	3.41E-05	10
GO:0051052	regulation of DNA metabolic process	3.15	0.71	4.68E-05	1.37E-03	1.11E-03	10
GO:0035315	hair cell differentiation	3.15	0.83	2.02E-04	4.10E-03	3.33E-03	10
GO:0035316	non-sensory hair organization	3.15	0.83	2.02E-04	4.10E-03	3.33E-03	10
GO:0048546	digestive tract morphogenesis	3.15	0.86	2.50E-04	4.76E-03	3.86E-03	10
GO:0007052	mitotic spindle organization	3.15	0.90	3.75E-04	6.11E-03	4.96E-03	10
GO:0007098	centrosome cycle	3.15	1.08	1.75E-03	1.95E-02	1.58E-02	10
GO:0016319	mushroom body development	3.15	1.10	2.03E-03	2.21E-02	1.79E-02	10
GO:0031023	microtubule organizing center organization	3.15	1.14	2.70E-03	2.67E-02	2.17E-02	10
GO:0007422	peripheral nervous system development	3.15	1.20	4.01E-03	3.59E-02	2.91E-02	10
GO:0048863	stem cell differentiation	3.15	1.20	4.01E-03	3.59E-02	2.91E-02	10
GO:0007548	sex differentiation	3.15	1.26	5.77E-03	4.74E-02	3.85E-02	10
GO:0016318	ommatidial rotation	2.84	0.53	2.03E-05	7.28E-04	5.91E-04	9
GO:0051304	chromosome separation	2.84	0.65	1.29E-04	2.93E-03	2.38E-03	9
GO:0098742	cell-cell adhesion via plasma-membrane adhesion molecules	2.84	0.73	3.45E-04	5.95E-03	4.84E-03	9
GO:0007143	female meiotic nuclear division	2.84	0.75	4.30E-04	6.85E-03	5.57E-03	9
GO:0035317	imaginal disc-derived wing hair organization	2.84	0.81	7.96E-04	1.10E-02	8.97E-03	9
GO:0007088	regulation of mitotic nuclear division	2.84	0.83	9.63E-04	1.29E-02	1.04E-02	9
GO:0008045	motor neuron axon guidance	2.84	0.90	1.64E-03	1.87E-02	1.52E-02	9
GO:0001655	urogenital system development	2.84	0.94	2.28E-03	2.39E-02	1.94E-02	9

GO:0035151	regulation of tube size, open tracheal system	2.84	0.94	2.28E-03	2.39E-02	1.94E-02	9
GO:0045787	positive regulation of cell cycle	2.84	0.94	2.28E-03	2.39E-02	1.94E-02	9
GO:0072001	renal system development	2.84	0.94	2.28E-03	2.39E-02	1.94E-02	9
GO:0006310	DNA recombination	2.84	0.98	3.09E-03	2.97E-02	2.41E-02	9
GO:0030010	establishment of cell polarity	2.84	1.02	4.12E-03	3.64E-02	2.95E-02	9
GO:0007156	homophilic cell adhesion via plasma membrane adhesion molecules	2.52	0.55	2.07E-04	4.15E-03	3.37E-03	8
GO:0030178	negative regulation of Wnt signaling pathway	2.52	0.57	2.74E-04	5.05E-03	4.10E-03	8
GO:2001251	negative regulation of chromosome organization	2.52	0.59	3.57E-04	5.95E-03	4.84E-03	8
GO:0035329	hippo signaling	2.52	0.61	4.60E-04	7.16E-03	5.82E-03	8
GO:0071897	DNA biosynthetic process	2.52	0.63	5.85E-04	8.71E-03	7.08E-03	8
GO:0035159	regulation of tube length, open tracheal system	2.52	0.69	1.14E-03	1.46E-02	1.19E-02	8
GO:0048859	formation of anatomical boundary	2.52	0.69	1.14E-03	1.46E-02	1.19E-02	8
GO:0007127	meiosis I	2.52	0.83	4.03E-03	3.59E-02	2.91E-02	8
GO:0033046	negative regulation of sister chromatid segregation	2.21	0.29	1.01E-05	3.93E-04	3.19E-04	7
GO:0051784	negative regulation of nuclear division	2.21	0.29	1.01E-05	3.93E-04	3.19E-04	7
GO:0051985	negative regulation of chromosome segregation	2.21	0.29	1.01E-05	3.93E-04	3.19E-04	7
GO:0007076	mitotic chromosome condensation	2.21	0.31	1.79E-05	6.54E-04	5.31E-04	7
GO:0042023	DNA endoreduplication	2.21	0.31	1.79E-05	6.54E-04	5.31E-04	7
GO:0007494	midgut development	2.21	0.37	7.47E-05	2.03E-03	1.65E-03	7
GO:0035330	regulation of hippo signaling	2.21	0.49	5.80E-04	8.71E-03	7.08E-03	7
GO:0045448	mitotic cell cycle, embryonic	2.21	0.51	7.62E-04	1.07E-02	8.72E-03	7
GO:0033045	regulation of sister chromatid segregation	2.21	0.53	9.86E-04	1.31E-02	1.06E-02	7
GO:0051983	regulation of chromosome segregation	2.21	0.55	1.26E-03	1.60E-02	1.30E-02	7
GO:0048619	embryonic hindgut morphogenesis	2.21	0.59	1.98E-03	2.16E-02	1.75E-02	7
GO:0007442	hindgut morphogenesis	2.21	0.67	4.33E-03	3.71E-02	3.01E-02	7
GO:0061525	hindgut development	2.21	0.67	4.33E-03	3.71E-02	3.01E-02	7
GO:0048645	animal organ formation	2.21	0.71	6.10E-03	4.94E-02	4.01E-02	7

GO:0006275	regulation of DNA replication	1.89	0.24	4.57E-05	1.36E-03	1.11E-03	6
GO:0006271	DNA strand elongation involved in DNA replication	1.89	0.26	8.03E-05	2.05E-03	1.67E-03	6
GO:0022616	DNA strand elongation	1.89	0.26	8.03E-05	2.05E-03	1.67E-03	6
GO:0006277	DNA amplification	1.89	0.35	4.64E-04	7.16E-03	5.82E-03	6
GO:0035277	spiracle morphogenesis, open tracheal system	1.89	0.35	4.64E-04	7.16E-03	5.82E-03	6
GO:0051302	regulation of cell division	1.89	0.41	1.23E-03	1.58E-02	1.28E-02	6
GO:0007062	sister chromatid cohesion	1.89	0.43	1.63E-03	1.87E-02	1.52E-02	6
GO:0016321	female meiosis chromosome segregation	1.89	0.43	1.63E-03	1.87E-02	1.52E-02	6
GO:0007443	Malpighian tubule morphogenesis	1.89	0.53	5.27E-03	4.43E-02	3.59E-02	6
GO:0061333	renal tubule morphogenesis	1.89	0.53	5.27E-03	4.43E-02	3.59E-02	6
GO:0032465	regulation of cytokinesis	1.58	0.20	2.09E-04	4.15E-03	3.37E-03	5
GO:0031577	spindle checkpoint	1.58	0.22	3.63E-04	5.95E-03	4.84E-03	5
GO:0033048	negative regulation of mitotic sister chromatid segregation	1.58	0.22	3.63E-04	5.95E-03	4.84E-03	5
GO:0045841	negative regulation of mitotic metaphase/anaphase transition	1.58	0.22	3.63E-04	5.95E-03	4.84E-03	5
GO:1902100	negative regulation of metaphase/anaphase transition of cell cycle	1.58	0.22	3.63E-04	5.95E-03	4.84E-03	5
GO:1905819	negative regulation of chromosome separation	1.58	0.22	3.63E-04	5.95E-03	4.84E-03	5
GO:2000816	negative regulation of mitotic sister chromatid separation	1.58	0.22	3.63E-04	5.95E-03	4.84E-03	5
GO:0007157	heterophilic cell-cell adhesion via plasma membrane cell adhesion molecules	1.58	0.24	5.89E-04	8.71E-03	7.08E-03	5
GO:0045839	negative regulation of mitotic nuclear division	1.58	0.24	5.89E-04	8.71E-03	7.08E-03	5
GO:0050000	chromosome localization	1.58	0.24	5.89E-04	8.71E-03	7.08E-03	5
GO:0007307	eggshell chorion gene amplification	1.58	0.31	1.90E-03	2.09E-02	1.69E-02	5
GO:0016339	calcium-dependent cell-cell adhesion via plasma membrane cell adhesion molecules	1.58	0.35	3.52E-03	3.25E-02	2.64E-02	5
GO:0044719	regulation of imaginal disc-derived wing size	1.58	0.35	3.52E-03	3.25E-02	2.64E-02	5
GO:0007094	mitotic spindle assembly checkpoint	1.26	0.20	2.62E-03	2.62E-02	2.13E-02	4
GO:0007100	mitotic centrosome separation	1.26	0.20	2.62E-03	2.62E-02	2.13E-02	4
GO:0014019	neuroblast development	1.26	0.20	2.62E-03	2.62E-02	2.13E-02	4

GO:0035223	leg disc pattern formation	1.26	0.20	2.62E-03	2.62E-02	2.13E-02	4
GO:0051054	positive regulation of DNA metabolic process	1.26	0.20	2.62E-03	2.62E-02	2.13E-02	4
GO:0071173	spindle assembly checkpoint	1.26	0.20	2.62E-03	2.62E-02	2.13E-02	4
GO:0071174	mitotic spindle checkpoint	1.26	0.20	2.62E-03	2.62E-02	2.13E-02	4
GO:0040034	regulation of development, heterochronic	1.26	0.22	3.91E-03	3.55E-02	2.88E-02	4
GO:0048864	stem cell development	1.26	0.22	3.91E-03	3.55E-02	2.88E-02	4
GO:0007449	proximal/distal pattern formation, imaginal disc	1.26	0.24	5.57E-03	4.61E-02	3.75E-02	4
GO:0036098	male germ-line stem cell population maintenance	1.26	0.24	5.57E-03	4.61E-02	3.75E-02	4
