# Supplementary material for: Transcriptomic changes during caste development through social interactions in the termite Zootermopsis nevadensis
Source: Ecol Evol. 2019 Feb 23;9(6):3446–56. doi: 10.1002/ece3.4976 (PMC6434549; doi:10.1002/ece3.4976)
Supplement: Supplementary file 22 [file ECE3-9-3446-s022.pdf]

Table S20. The enriched GO terms in the No. 2 larva compared with the No. 1 larva at Day 3.

ID	Description	% in caste-DEG	% in all	pvalue	p.adjust	qvalue	Count
GO:0055114	oxidation-reduction process	14.85	6.25	4.51E-09	6.37E-06	5.84E-06	49
GO:0006811	ion transport	12.73	5.88	9.44E-07	2.22E-04	2.04E-04	42
GO:0006082	organic acid metabolic process	11.52	5.17	1.58E-06	2.49E-04	2.28E-04	38
GO:0043436	oxoacid metabolic process	11.52	5.17	1.58E-06	2.49E-04	2.28E-04	38
GO:0055085	transmembrane transport	11.52	5.37	4.15E-06	5.86E-04	5.38E-04	38
GO:1901135	carbohydrate derivative metabolic process	11.52	6.80	7.43E-04	3.39E-02	3.11E-02	38
GO:0019752	carboxylic acid metabolic process	11.21	4.78	6.10E-07	1.72E-04	1.58E-04	37
GO:0042335	cuticle development	6.06	2.22	2.85E-05	2.87E-03	2.64E-03	20
GO:0006820	anion transport	5.76	2.18	7.22E-05	5.67E-03	5.20E-03	19
GO:0006520	cellular amino acid metabolic process	5.76	2.42	3.10E-04	1.82E-02	1.67E-02	19
GO:0048066	developmental pigmentation	4.85	1.65	7.23E-05	5.67E-03	5.20E-03	16
GO:0043473	pigmentation	4.85	1.69	9.83E-05	7.31E-03	6.70E-03	16
GO:0015711	organic anion transport	4.55	1.63	2.24E-04	1.38E-02	1.26E-02	15
GO:0006040	amino sugar metabolic process	4.24	0.77	6.41E-08	3.02E-05	2.77E-05	14
GO:0006022	aminoglycan metabolic process	4.24	0.96	1.30E-06	2.49E-04	2.28E-04	14
GO:1901605	alpha-amino acid metabolic process	4.24	1.40	1.51E-04	1.07E-02	9.80E-03	14
GO:0006030	chitin metabolic process	3.94	0.59	1.15E-08	8.09E-06	7.42E-06	13
GO:1901071	glucosamine-containing compound metabolic process	3.94	0.71	1.72E-07	6.07E-05	5.57E-05	13
GO:0048067	cuticle pigmentation	3.33	0.73	1.40E-05	1.52E-03	1.39E-03	11
GO:0046148	pigment biosynthetic process	3.33	1.04	4.44E-04	2.51E-02	2.30E-02	11
GO:0015849	organic acid transport	3.33	1.10	7.44E-04	3.39E-02	3.11E-02	11
GO:0046942	carboxylic acid transport	3.33	1.10	7.44E-04	3.39E-02	3.11E-02	11
GO:1901617	organic hydroxy compound biosynthetic process	3.03	0.69	5.01E-05	4.72E-03	4.33E-03	10
GO:0009187	cyclic nucleotide metabolic process	3.03	0.71	6.60E-05	5.67E-03	5.20E-03	10
GO:0009190	cyclic nucleotide biosynthetic process	2.73	0.65	1.76E-04	1.13E-02	1.04E-02	9

GO:0052652	cyclic purine nucleotide metabolic process	2.73	0.65	1.76E-04	1.13E-02	1.04E-02	9
GO:0046189	phenol-containing compound biosynthetic process	2.12	0.26	7.00E-06	8.99E-04	8.24E-04	7
GO:0009072	aromatic amino acid family metabolic process	2.12	0.29	1.32E-05	1.52E-03	1.39E-03	7
GO:0044550	secondary metabolite biosynthetic process	2.12	0.49	7.40E-04	3.39E-02	3.11E-02	7
GO:0006026	aminoglycan catabolic process	1.82	0.35	5.77E-04	3.13E-02	2.87E-02	6
GO:0019933	cAMP-mediated signaling	1.82	0.39	1.13E-03	4.97E-02	4.56E-02	6
GO:0007189	adenylate cyclase-activating G-protein coupled receptor signaling pathway	1.52	0.24	7.09E-04	3.39E-02	3.11E-02	5
