# Supplementary material for: Transcriptomic changes during caste development through social interactions in the termite Zootermopsis nevadensis
Source: Ecol Evol. 2019 Feb 23;9(6):3446–56. doi: 10.1002/ece3.4976 (PMC6434549; doi:10.1002/ece3.4976)
Supplement: Supplementary file 23 [file ECE3-9-3446-s023.docx]

Figure S1. The diagrams showing the numbers of caste-biased genes with ages obtained by ANOVA-like test with GLM analysis. The numbers in a graph show those of differentially expressed genes (DEGs) in soldier-destined (No. 1) and worker-destined (No. 2) larvae with ages.

Figure S2. The diagrams showing the numbers of enriched GO terms. The numbers in a graph show those of upregulated GO terms in each pairwise comparison.
